# Sustained improvements in brain health and metabolic markers 24 months following bariatric surgery

**DOI:** 10.1093/braincomms/fcae336

**Published:** 2024-10-04

**Authors:** Marianne Legault, Mélissa Pelletier, Amélie Lachance, Marie-Ève Lachance, Yashar Zeighami, Marie-Frédérique Gauthier, Sylvain Iceta, Laurent Biertho, Stephanie Fulton, Denis Richard, Alain Dagher, André Tchernof, Mahsa Dadar, Andréanne Michaud

**Affiliations:** Quebec Heart and Lung Institute Research Centre, Université Laval, Quebec City, Quebec, Canada, G1V 4G5; Centre Nutrition, Santé et Societé (NUTRISS), Université Laval, Quebec City, Quebec, Canada, G1V 4L3; Quebec Heart and Lung Institute Research Centre, Université Laval, Quebec City, Quebec, Canada, G1V 4G5; Quebec Heart and Lung Institute Research Centre, Université Laval, Quebec City, Quebec, Canada, G1V 4G5; Centre Nutrition, Santé et Societé (NUTRISS), Université Laval, Quebec City, Quebec, Canada, G1V 4L3; Quebec Heart and Lung Institute Research Centre, Université Laval, Quebec City, Quebec, Canada, G1V 4G5; Centre Nutrition, Santé et Societé (NUTRISS), Université Laval, Quebec City, Quebec, Canada, G1V 4L3; Douglas Research Center, McGill University, Montreal, Quebec, Canada, H4H 1R3; Quebec Heart and Lung Institute Research Centre, Université Laval, Quebec City, Quebec, Canada, G1V 4G5; Quebec Heart and Lung Institute Research Centre, Université Laval, Quebec City, Quebec, Canada, G1V 4G5; General Surgery Department, Quebec Heart and Lung Institute, Université Laval, Quebec City, Quebec, Canada, G1V 4G5; CRCHUM and Montreal Diabetes Research Center, Montreal University, Montreal, Quebec, Canada, H2X 0A9; Quebec Heart and Lung Institute Research Centre, Université Laval, Quebec City, Quebec, Canada, G1V 4G5; Montreal Neurological Institute, McGill University, Montreal, Quebec, Canada, H3A 2B4; Quebec Heart and Lung Institute Research Centre, Université Laval, Quebec City, Quebec, Canada, G1V 4G5; Douglas Research Center, McGill University, Montreal, Quebec, Canada, H4H 1R3; Quebec Heart and Lung Institute Research Centre, Université Laval, Quebec City, Quebec, Canada, G1V 4G5; Centre Nutrition, Santé et Societé (NUTRISS), Université Laval, Quebec City, Quebec, Canada, G1V 4L3

**Keywords:** obesity, bariatric surgery, weight loss, grey matter, white matter

## Abstract

Obesity and its metabolic complications are associated with lower grey matter and white matter densities, whereas weight loss after bariatric surgery leads to an increase in both measures. These increases in grey and white matter density are significantly associated with post-operative weight loss and improvement of the metabolic/inflammatory profiles. While our recent studies demonstrated widespread increases in white matter density 4 and 12 months after bariatric surgery, it is not clear if these changes persist over time. The underlying mechanisms also remain unknown. In this regard, numerous studies demonstrate that the enlargement or hypertrophy of mature adipocytes, particularly in the visceral fat compartment, is an important marker of adipose tissue dysfunction and obesity-related cardiometabolic abnormalities. We aimed (i) to assess whether the increases in grey and white matter densities previously observed at 12 months are maintained 24 months after bariatric surgery; (ii) to examine the association between these structural brain changes and adiposity and metabolic markers 24 months after bariatric surgery; and (iii) to examine the association between abdominal adipocyte diameter at the time of surgery and post-surgery grey and white matter densities changes. Thirty-three participants undergoing bariatric surgery were recruited. Grey and white matter densities were assessed from T1-weighted magnetic resonance imaging scans acquired prior to and 4, 12 and 24 months post-surgery using voxel-based morphometry. Omental and subcutaneous adipose tissue samples were collected during the surgical procedure. Omental and subcutaneous adipocyte diameters were measured by microscopy of fixed adipose tissue samples. Linear mixed-effects models were performed controlling for age, sex, surgery type, initial body mass index, and initial diabetic status. The average weight loss at 24 months was 33.6 ± 7.6%. A widespread increase in white matter density was observed 24 months post-surgery mainly in the cerebellum, brainstem and corpus callosum (*P* < 0.05, false discovery rate) as well as some regions in grey matter density. Greater omental adipocyte diameter at the time of surgery was associated with greater changes in total white matter density at 24 months (*P* = 0.008). A positive trend was observed between subcutaneous adipocyte diameter at the time of surgery and changes in total white matter density at 24 months (*P* = 0.05). Our results show prolonged increases in grey and white matter densities up to 24 months post-bariatric surgery. Greater preoperative omental adipocyte diameter is associated with greater increases in white matter density at 24 months, suggesting that individuals with excess visceral adiposity might benefit the most from surgery.

## Teaser

Legault *et al*. report extended increases in white matter density up to 24 months post-bariatric surgery, which are related to metabolic and adiposity improvements. Greater preoperative omental adipocyte diameter is associated with a greater increase in white matter density post-surgery, suggesting that individuals with higher abdominal adiposity might benefit the most from surgery.

## Graphical Abstract

**Graphical Abstract:**
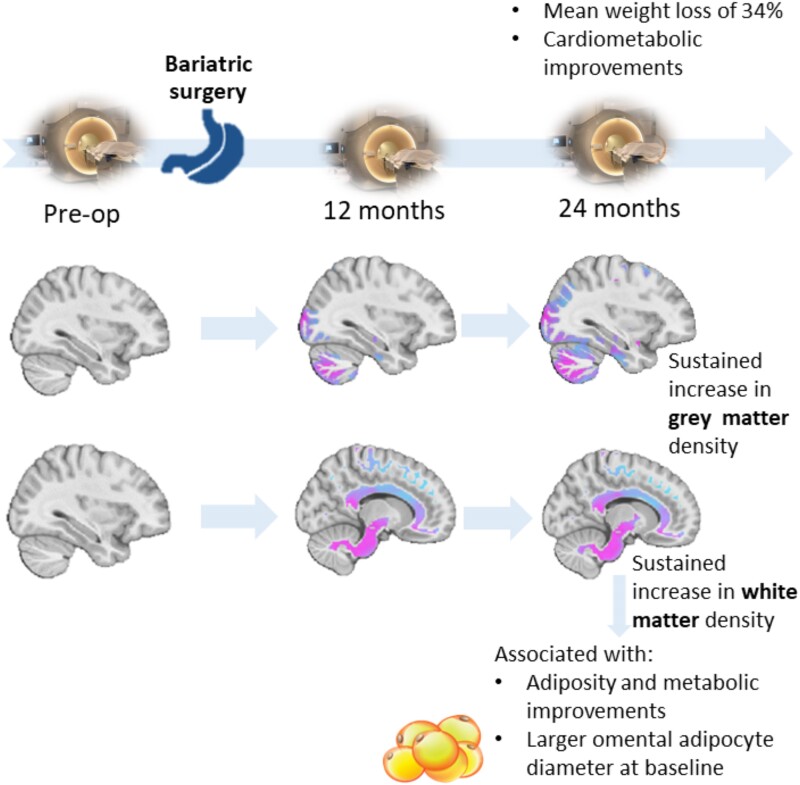

## Introduction

Excessive accumulation of adipose tissue in the body, especially in the visceral fat compartment (mesentery and greater omentum), is strongly associated with cardiometabolic alterations, including insulin resistance, dyslipidemia, hypertension and chronic, low-grade inflammation.^1,2^ Recent studies have highlighted that dysfunction in subcutaneous adipose tissue, limiting its capacity for expansion, can lead to spill over into the system, resulting in ectopic fat accumulation in other organs.^3^ This mechanism is a major factor explaining the link between visceral obesity and cardiometabolic alterations.^3^ Numerous studies demonstrate that the enlargement or hypertrophy of mature adipocytes, particularly in the visceral fat compartment, is an important marker of adipose tissue dysfunction^4^ and obesity-related cardiometabolic abnormalities.^5^

In addition to these cardiometabolic alterations, overall and abdominal obesity in midlife are risk factors for cognitive impairment and dementia.^6-8^ Previous cross-sectional and longitudinal magnetic resonance imaging (MRI) studies demonstrated that obesity is associated with reduced grey matter (GM) volume and lower cortical thickness, mainly in frontotemporal regions^9-13^. These results were corroborated in a recent review by Li *et al*.,^14^ which also reported that obesity, as measured by body mass index (BMI), was negatively associated with white matter (WM) volume. Other studies have also shown that adiposity is related to increased WM hyperintensities, which were in turn related to lower cortical thickness and diminished GM volume.^15,16^ A meta-analysis by Daoust *et al*.^17^ highlighted that obesity is associated with reduced WM integrity in a tract linking frontal areas involved in executive function. These alterations in brain integrity might explain the association between abdominal obesity, accelerating brain ageing and cognitive impairment. Even if the exact mechanisms underlying these obesity-related brain abnormalities are poorly characterized, there is an increasing number of studies showing that abdominal obesity-related cardiometabolic alterations, such as chronic, low-grade inflammation, hypertension and insulin resistance, might have negative effects on cerebral GM and WM integrity by disrupting cerebral blood flow,^18^ leading to cerebral hypoperfusion and cognitive impairment.^15,18^

Bariatric surgery is an interesting model to examine the impact of marked weight loss and cardiometabolic improvements on brain health in a longitudinal setting.^19-21^ The first results of our brain MRI study reported rapid and important structural and functional brain changes 4 and 12 months after bariatric surgery.^22^ We observed widespread increases in WM and GM densities^22^ as well as resting neural activity^23^ 12 months after bariatric surgery, suggesting a global effect on brain health. Similar findings were also reported by other groups, with some observing changes as early as 1 month post-surgery.^24-28^ In parallel to the increase in GM density, we found an increase in spontaneous neural activity following surgery, assessed by the fractional amplitude of low-frequency fluctuations (fALFF) signal.^23^ Another study has also found that a reduction in BMI was correlated with increased functional connectivity in specific brain regions in the first months following surgery.^29^ These structural and functional increases were significantly associated with the magnitude of weight loss and metabolic improvements induced by bariatric surgery, suggesting that it may lead to a resolution of adiposity-related brain abnormalities along with widespread improvements in metabolism.^22,23^ These improvements could potentially result from the reduction of inflammatory markers, improvements in cerebral blood flow and changes in gastrointestinal appetite-regulating hormones after surgery, such as insulin, ghrelin, glucagon-like peptide-1 (GLP-1) and leptin, that may impact the brain.^14,30,31^

Despite the growing evidence showing seemingly beneficial brain structural changes after bariatric surgery, it remains unclear if these changes persist longer than 12 months.^22-25^ One recent study examined changes up to 24 months post-surgery, reporting reductions in GM volume with no change in hippocampal and WM volume.^32^ However, this study did not assess changes at a voxel level. Additionally, the mechanisms underlying these changes are still unknown. The objectives of the present study were (i) to assess whether the increases in GM and WM densities previously observed at 12 months are maintained 24 months after surgery; (ii) to examine the association between these structural brain changes and markers of adiposity, metabolism and inflammation 24 months post-surgery; and (iii) to examine the association between abdominal adipocyte diameter at the time of surgery and post-surgery changes in GM and WM densities. By focusing on abdominal adipoctyte diameter, we aimed to explore a potentially more sensitive indicator of the metabolic processes that might affect brain structure. This measure was chosen because it is a well-documented marker of metabolic dysfunction, which we hypothesized would significantly correlate with changes in brain integrity. Finally, to verify that the changes in GM and WM densities observed after bariatric surgery are not due to any confounders related to the repetition of the MRI session and are related to the weight loss-induced bariatric surgery, a separate group of participants matched with the surgery group for initial age and BMI underwent two scanning sessions before the surgery.

## Materials and methods

### Participant recruitment

Patients with severe obesity (*n* = 33, 24 women, 9 men, mean age = 46.9 ± 7.6 years, mean BMI = 44.0 ± 4.3 kg/m²) scheduled to receive bariatric surgery at *Institut universitaire de cardiologie et pneumologie de Québec - Université Laval* (IUCPQ-UL) were recruited. Participants were tested before and 4, 12 and 24 months after surgery. To make sure that the brain changes observed after bariatric surgery were not due to any confounders related to the repetition of the MRI session, we recruited a group of 19 participants (13 women, 6 men, mean age = 43.8 ± 10.2 years, mean BMI = 41.9 ± 3.8 kg/m²) who were tested two times prior to surgery, approximately 4 and 1 month before surgery. Inclusion criteria were the following: (i) women or men with a BMI ≥35 kg/m² who require gastro-intestinal surgery and who meet the National Institutes of Health Guidelines for bariatric surgery^33^ and (ii) age between 18 and 60 years. Exclusion criteria were the following: (i) BMI < 35 kg/m²; (ii) age under 18 or over 60 years; (iii) any uncontrolled medical, surgical, neurological or psychiatric condition; (iv) liver cirrhosis or albumin deficiency; (v) any medication that may affect the central nervous system; (vi) pregnancy; (vii) substance or alcohol abuse; (viii) previous gastric, oesophageal, brain or bariatric surgery; (ix) gastrointestinal inflammatory disease or gastrointestinal ulcers; (x) severe food allergy; and (xi) contraindications to MRI (implanted medical device, metal fragment in body or claustrophobia). The study was approved by the Research Ethics Committee of the *Centre de recherche de l’IUCPQ-UL* (CRIUCPQ-UL), and all participants provided written informed consent to participate in the study.

### Surgical procedures

Participants underwent sleeve gastrectomy, Roux-en-Y gastric bypass or biliopancreatic diversion with a duodenal switch. All surgeries were performed at the IUCPQ-UL. Most participants underwent sleeve gastrectomy (*n* = 22), consisting of a 250 cm^3^ vertical gastrectomy starting 5–7 cm from the pylorus to the Hiss angle, using a 34–44 French Bougie for guidance, to create a gastric tube.^34^ The greater curvature and fundus of the stomach were removed. Seven participants underwent a laparoscopic Roux-en-Y gastric bypass in which a proximal gastric pouch of 30–50 cm^3^ is created and anastomosed to the proximal small bowel by bypassing the first 100 cm and bringing a 100 cm alimentary limb on the gastric pouch.^23^ Four participants underwent biliopancreatic diversion with duodenal switch, which is a mixed surgery combining restrictive and malabsorptive mechanisms by creating a 250 cm^3^ vertical sleeve gastrectomy and duodeno–ileal anastomosis 100 cm from ileocecal valve.^35^

### Study design and experimental procedures

The study design has been described in detail by Michaud *et al*.^22^ Briefly, participants were asked to attend four sessions: prior to and 4, 12 and 24 months after surgery. These time points align with routine care post-bariatric surgery at our institution, facilitating both recruitment and retention efforts. At each session, all the participants had a complete physical and medical evaluation (blood pressure, anthropometry, i.e. weight by bioimpedance, height, waist, neck and hip circumference with standard procedures^22^) and a brain MRI session. BMI was calculated from weight and height and total weight loss was calculated by subtracting follow-up weight from baseline weight. Excess weight loss was calculated using preoperative weight, post-operative weight and ideal body weight for a BMI of 23 kg/m^2^ as previously used.^36^ Fasting blood samples were taken on the morning of each visit. We used ethylenediaminetetraacetic-coated tubes or serum clot activator tubes. All samples were immediately placed at 4°C and then centrifuged and stored at −80°C. The plasma lipid profile [cholesterol, high-density lipoproteins (HDL), low-density lipoproteins (LDL) and triglyceride levels], glucose homeostasis (plasma insulin and glucose levels) and high-sensitivity C-reactive protein (CRP) levels were measured by the Biochemistry Department of the IUCPQ-UL. The homeostasis model assessment insulin resistance (HOMA-IR) index was calculated using this formula: [fasting glucose (mmol/L) × fasting insulin (pmol/L)]/135.

### T1-weighted MRI acquisition

MRI acquisition was conducted in the morning, and participants were asked to fast 12 h before the scanning session. One hour before the MRI session, participants were given a standardized beverage meal (237 ml, 240 kcal, Boost Original, Nestle Health Science) to control for hunger levels. They were asked to drink it within a 5 min period. Hunger levels were then assessed using a visual analogue scale (VAS) with ratings obtained before and after the MRI session. The MRI protocol included anatomical T1-weighted three-dimensional (3D) turbo field echo images, resting state functional MRI (fMRI) and a task state fMRI for food-cue reactivity. In the current study, only results from anatomical images are presented. T1-weighted three-dimensional (3D) turbo field echo images were acquired at each visit using a 3T whole-body MRI scanner (Philips, Ingenia, Philips Medical Systems) equipped with a 32-channel head coil at the CRIUCPQ-UL. The following parameters were used: 176 sagittal 1.0 mm slices, repetition time/echo time (TR/TE) = 8.1/3.7 ms, field of view (FOV) = 240 × 240 mm^2^ and voxel size = 1 × 1 × 1 mm.

### Voxel-based morphometry measurements

GM and WM densities were assessed from each T1-weighted MRI using a standard VBM pipeline.^10^ The preprocessing steps were the following: (i) image denoising^37^; (ii) intensity non-uniformity correction^38^; and (iii) image intensity normalization into range (0–100) using histogram matching. Images were then first linearly and then non-linearly registered to an average brain template (MNI ICBM152) as part of the ANIMAL software^39^ and segmented into GM, WM and cerebrospinal fluid images. These steps remove global differences in the size and shape of individual brains and transform individual GM or WM density maps to the standardized MNI ICBM152 template space. VBM analysis was performed using MNI/minc tools (http://www.bic.mni.mcgill.ca/ServicesSoftware/MINC) to generate GM and WM density maps representing the local GM/WM concentration per voxel. To avoid the overlap between GM and WM signals in their border due to a combination of partial volume effect and smoothing of the maps, we removed three voxels on the border of the GM and WM regions.

### Adipocyte diameter

Subcutaneous (SC) and omental (OM) adipose tissue samples (*n* = 29) were collected during the surgical procedure at the site of the incision (lower abdomen) and at the greater omentum, respectively. Adipose tissue samples were fixed as previously described.^40^ Adipocyte size was measured using automated segmentation with ImageJ (https://imagej.net) based on the method of Maguire *et al*.^41^ Whole slides were first digitized using a Zeiss Axio Scan.Z1 in brightfield at 20× magnification. The images were then converted into black and white 8-bit images, and the area of each adipocyte cell was measured. The mean areas were converted in diameter in micrometres. Samples with under 200 cells counted (*n* = 14) were manually validated to assess the quality of the tissue. Samples with extreme tissue damage (*n* = 3) were excluded.

### Statistical analysis

Repeated-measures ANOVA or Student’s *t*-test was used to compare the clinical characteristics of participants across each session.

Linear mixed-effects models (Model I) were used to assess VBM changes in GM and WM following bariatric surgery (Objective 1). The covariates included age, sex, baseline BMI, baseline diabetic status and surgery type, with session (baseline, 4, 12 and 24 months) as a fixed effect and subject as a categorical random variable.

***Model I***: VBM GM or WM ∼Session + Age _baseline_ + Sex + BMI _baseline_ + Diabetic status _baseline_ + Surgery type + (1|Subject)

To control for the potential effect of surgery type, a similar model was used to evaluate VBM changes in GM and WM in the sleeve gastrectomy subset (*N* = 26). This model included age, sex, baseline BMI and baseline diabetic status as covariates, session (baseline, 4, 12 and 24 months) as a fixed effect and subject as a random variable.

To assess VBM changes in GM and WM following bariatric surgery while controlling for medication intake, linear mixed-effects models (Model II) were used. This model included age, sex, baseline BMI, baseline diabetic status, surgery type and medication intakes for diabetes, hypertension and dyslipidemia as covariates, session (baseline, 4, 12 and 24 months) as a fixed effect and subject as a random variable.

***Model II***: VBM GM or WM ∼Session + Age _baseline_ + Sex + BMI _baseline_ + Diabetic status _baseline_ + Surgery type + T2D medication + antihypertensive medication + lipid-lowering medication + (1|Subject)

To examine the associations with adiposity, metabolic and inflammatory variables and surgery-induced brain changes in GM or WM density (Objective 2), the automated anatomical labeling Atlas^42^ was used to extract average regional VBM GM values and similarly, the Atlas from Yeh *et al*.^43^ was used to extract regional VBM WM values for each participant. Mean GM and WM densities were extracted across all the participants. Linear mixed-effects models (Model III) were used to assess the associations between post-surgery changes in GM or WM density and changes in adiposity, metabolic and inflammatory variables with age at baseline and sex as covariates to ensure the stability of the findings.

***Model III***: VBM GM or WM ∼ Cardiometabolic variables + Age _baseline_ + Sex + (1|Subject)

Linear mixed-effects models (Model IV) were also performed to examine the associations between OM or SC adipocyte diameter at the time of surgery and changes in total GM or WM density (Objective 3), with age at baseline and sex as covariates and subject ID as a categorical random effect.

***Model IV*:** VBM GM or WM ∼ OM or SC adipocyte diameter _baseline_ + Age _baseline_ + Sex + (1|Subject)

The mixed-effects model estimates are represented by *t*-statistics in the results section. The VBM results were corrected for multiple comparisons using the false discovery rate (FDR) controlling technique (significance threshold of *P* < 0.05). Spearman correlations were performed to examine the associations between OM and SC adipocyte diameters at the time of surgery and metabolic/adiposity variables. Bonferroni correction was applied for multiple comparisons. To control for re-test effect (Objective 4), paired *t*-tests were used to compare data between the two sessions prior to surgery. Statistical analyses were performed with JMP software version 14 (SAS Institute Inc., Cary, NC, USA) and MATLAB software version R2021a (Natick, Massachusetts: The MathWorks Inc., USA).

## Results

### Clinical characteristics of participants

The clinical characteristics of participants are shown in Table 1. Participants were mainly women (73%), with a mean age at baseline of 46.9 ± 7.6 years. Ten participants were treated for Type 2 diabetes prior to surgery (3 with insulin and 7 with oral hypoglycaemic agents), 18 were treated for hypertension, and 9 were treated for dyslipidemia. As expected, BMI, waist circumference and neck circumference all significantly decreased after the surgery (*P* < 0.0001).

**Table 1:** Characteristics of participants at baseline, 4, 12 and 24 months after bariatric surgery

	Baseline	4 months	12 months	24 months	*P*-value
*N*	33	33	33	33	
Sex (F:M)	24:9				
Age (years)	46.9 ± 7.6	47.4 ± 7.5	48.1 ± 7.6	49.1 ± 7.7	
Diabetic (Y:N)	10:23				
BMI (kg/m^2^)	44.0 ± 4.3	34.7 ± 3.7	29.3 ± 3.6	29.2 ± 4.0	**<0.0001**; F = 104.1507
*Type of surgery*					
SG	22				
RYGB	7				
BPD-DS	4				
Waist circumference (cm)	130.8 ± 10.6	113.2 ± 9.2	101.2 ± 11.5	98.9 ± 11.2	**<0.0001**; F = 62.7222
Neck circumference (cm)	41.5 ± 3.6	36.4 ± 3.4	34.9 ± 2.8	34.6 ± 3.0	**<0.0001**; F = 26.2495
EWL (%)	-	45.4 ± 8.5	71.1 ± 13.3	69.5 ± 13.8	**<0.0001**; F = 307.2727
TWL (%)	-	21.2 ± 3.4	33.3 ± 6.2	33.6 ± 7.6	**<0.0001**; F = 311.7833
Systolic blood pressure (mmHg)	142.3 ± 13.8	123.4 ± 14.6	120.0 ± 14.5	115.6 ± 11.6	**<0.0001**; F = 22.7247
Diastolic blood pressure (mmHg)^a^	85.5 ± 9.3	75.2 ± 11.1	72.2 ± 9.8	68.8 ± 9.4	**<0.0001**; F = 17.5106
Fasting glucose (mmol/L)	6.5 ± 1.6	5.2 ± 1.2	4.8 ± 0.8	5.0 ± 0.8	**<0.0001**; F = 14.6839
Fasting insulin (pmol/L)	175.6 ± 105.8	61.7 ± 36.4	42.2 ± 23.8	44.1 ± 22.2	**<0.0001**; F = 36.6179
HOMA-IR index	8.8 ± 6.1	2.5 ± 2.2	1.5 ± 1.2	1.7 ± 0.9	**<0.0001**; F = 31.5851
Total cholesterol (mmol/L)^a^	4.4 ± 0.9	4.0 ± 1.2	4.1 ± 0.9	4.3 ± 0.9	0.2885; F = 1.2685
LDL cholesterol (mmol/L)	2.5 ± 0.9	2.5 ± 1.3	2.3 ± 0.9	2.3 ± 0.7	0.6880; F = 0.4927
HDL cholesterol (mmol/L)^a^	1.2 ± 0.3	1.2 ± 0.3	1.4 ± 0.3	1.5 ± 0.3	**<0.0001**; F = 10.6713
Triglycerides (mmol/L)	1.6 ± 0.7	1.4 ± 0.9	1.0 ± 0.3	1.0 ± 0.5	**0.0010**; F = 5.7539
CRP (mg/L)	5.53 ± 2.72	3.50 ± 3.84	0.89 ± 0.91	1.70 ± 3.54	**0.0041**; F = 4.9377
OM adipocyte cell diameter (μm)	62.7 ± 8.9				
SC adipocyte cell diameter (μm)	68.9 ± 7.9				
Hunger levels before the MRI session (0–10)	4.3 ± 2.7	3.0 ± 2.7	4.1 ± 2.7	3.6 ± 2.7	0.2150; F = 1.5106
Hunger levels after the MRI session (0–10)	6.8 ± 2.2	5.8 ± 2.4	6.9 ± 2.1	6.9 ± 1.9	0.1149; F = 2.0182

Results are presented as mean ± SD, with significant *P*-values in bold. F, female; M, male; Y, yes; N, no; BMI, body mass index; SG, sleeve gastrectomy; RYGB, Roux-en-Y gastric bypass; BPD-DS, biliopancreatic diversion with duodenal switch; EWL, excess weight loss; TWL, total weight loss; HOMA-IR, homeostatic model assessment for insulin resistance; LDL, low-density lipoprotein; HDL, high-density lipoprotein; CRP, C-reactive protein; OM, omental; SC, subcutaneous.

Repeated-measures ANOVA comparing baseline, 4, 12 and 24 months post-surgery sessions.

^a^Significant difference between 12 and 24 months (*P* < 0.05).

Most participants underwent sleeve gastrectomy. The mean total weight loss 24 months after surgery was 33.6 ± 7.6%. All metabolic and inflammatory markers significantly improved after the surgery [i.e. a decrease of systolic and diastolic blood pressure, fasting glucose and fasting insulin, and CRP levels and HOMA-IR index, as well as most lipid profile markers (i.e. HDL cholesterol and triglycerides)] as observed in Table 1. Only the total cholesterol and LDL cholesterol did not improve significantly. Interestingly, no significant difference was observed in the mean BMI, total weight loss and the improvement in most metabolic markers between 12 and 24 months post-surgery (*P* > 0.05, not shown in Table 1). There was a significant difference in diastolic blood pressure, which decreased from 12 to 24 months, and in total cholesterol and HDL cholesterol values, which increased over the same period (*P* < 0.05, not shown in Table 1). Hunger levels prior to and after the MRI session were similar at baseline, 4 months, 12 months and 24 months. Hunger levels after the MRI session were consistently higher compared to hunger levels before the MRI session across all visits (*P* < 0.0001).

### Effect of bariatric surgery on WM and GM density

**Figure 1:**
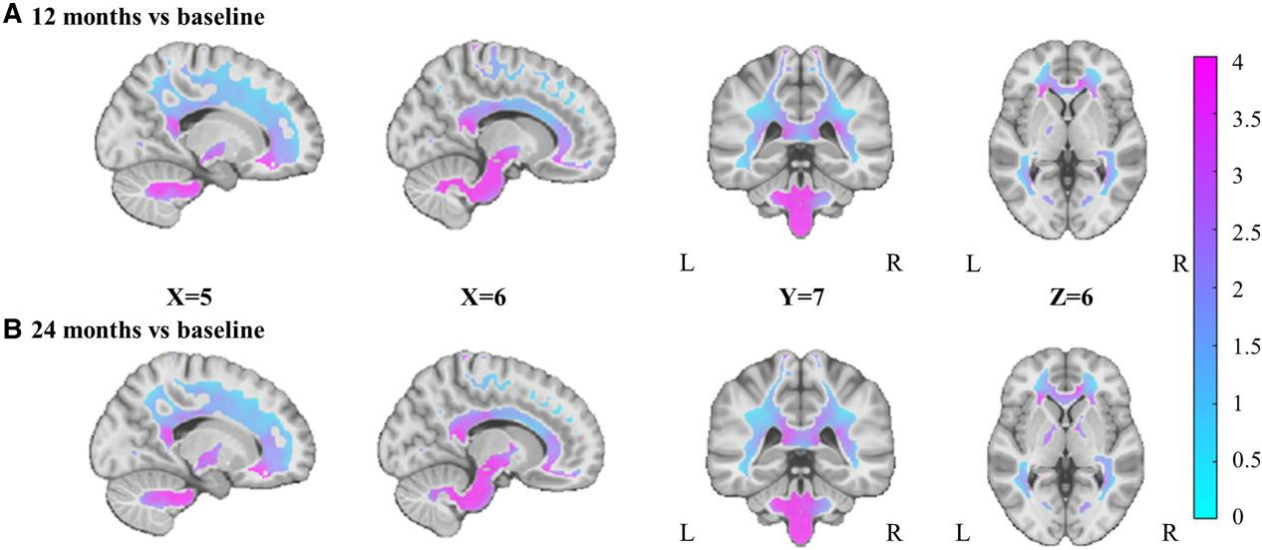
**Changes in white matter (WM) density (A) 12 and (B) 24 months post-surgery compared to baseline (*n* = 33).** The figure shows the *t*-value maps from the voxel-wise mixed-effects models for the WM regions that were significant after whole-brain FDR correction (*P* < 0.05), correcting for sex, age, BMI and diabetic status at baseline, as well as surgery type. The colours show higher positive *t*-values (in pink) or neutral *t*-values (in light blue). L, left; R, right.

**Figure 2:**
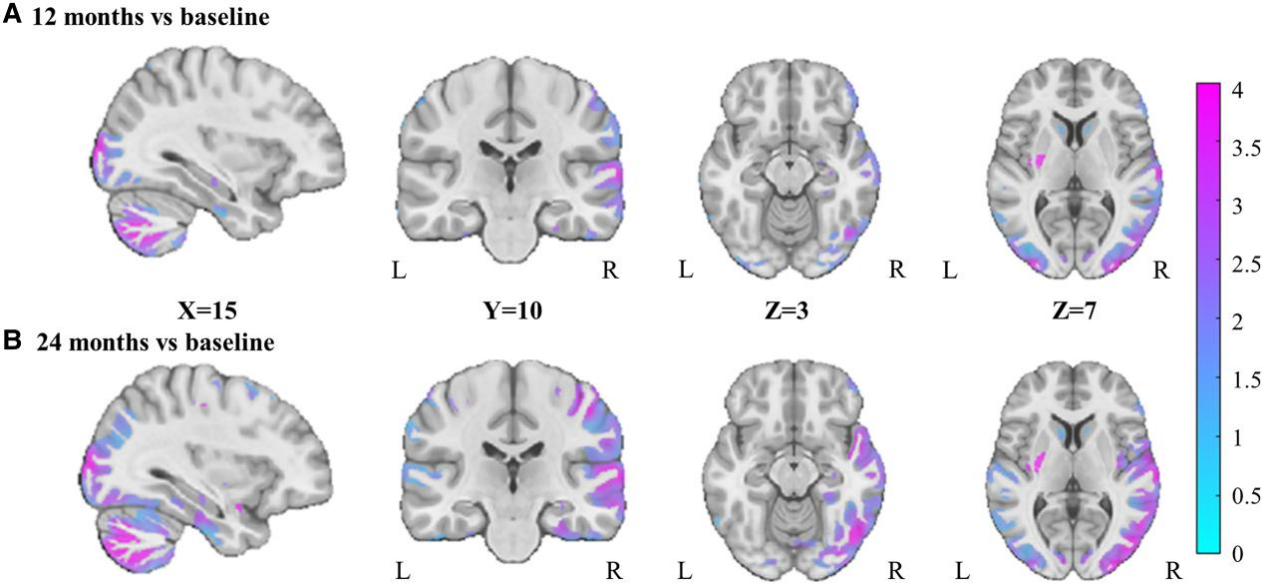
**Changes in grey matter (GM) density (A) 12 and (B) 24 months post-surgery compared to baseline (*n* = 33).** The figure shows the *t*-value maps from the voxel-wise mixed-effects models for the GM regions that were significant after whole-brain FDR correction (*P* < 0.05), correcting for sex, age, BMI and diabetic status at baseline, as well as surgery type. The colours show higher positive *t*-values (in pink) or neutral *t*-values(in light blue). L, left; R, right.

As shown in Figs 1 and 2, changes of voxel-wise WM and GM density previously observed at 12 months post-surgery^22^ were still present at 24 months post-surgery. There was a widespread increase in WM density, mainly in the cerebellum, brainstem and corpus callosum (Fig. 1). For GM density, there was also a widespread but less extensive increase, mainly in the cerebellum, occipital and temporal cortex and postcentral gyrus (Fig. 2). No significant difference was found between the types of surgery. Furthermore, there was also no significant difference in WM or GM densities when comparing 12 and 24 months (Figs 1 and 2). The results remained similar when including medication intakes as covariates in the models (Model II) (Supplementary Fig. 1**)**. A secondary analysis, conducted only with participants who underwent sleeve gastrectomy, yielded results consistent with our primary analysis (Supplementary Fig. 2).

To make sure that the structural brain changes observed after bariatric surgery were not due to any confounders related to the repetition of the MRI session, a group of participants (*n* = 19) had two pre-surgery sessions. The clinical characteristics of these participants were similar to our main cohort (Supplementary Table 1). Participants were mainly women and the mean age at pre-surgery session 1 was 43.8 ± 10.2 years old. As expected, there was no significant difference in the BMI of these participants between the two pre-surgery sessions (*P* = 0.9703, Supplementary Table 1), and no significant changes were observed in the WM and GM densities.

### Associations between changes in WM and GM densities and different markers after bariatric surgery

Linear mixed-effect models (Model III) were used to assess the association between changes in WM or GM density and adiposity, metabolic or inflammatory measurements after bariatric surgery, accounting for age and sex. The adiposity markers used for the associations between changes in regional WM and GM densities were BMI, total weight loss and waist and neck circumferences. Post-surgery reduction in adiposity measurements and total weight loss were significantly associated with increased WM density in 18 out of 27 regions, including acoustic radiation, central tegmental tract, cingulum, central nerve, corticospinal tract, corticostriatal and corticothalamic pathways, extreme capsule, fornix, frontopontine tract, inferior cerebellar peduncle, lateral lemniscus and pariotopontine tract (Supplementary Table 2, *P* < 0.0017). Regarding GM density, out of 34 regions, only increases in cingulate, rectus, precuneus, paracentral and vermis regions were significantly associated (Supplementary Table 3, *P* < 0.0014) with the reduction of one or more adiposity markers (BMI, total weight, waist and neck circumferences). A negative association was observed between total weight loss and increased GM density in occipital and angular regions (Supplementary Table 3, *P* < 0.0014).

Supplementary Tables 2 and 3 show the associations between post-operative changes in metabolic or inflammatory markers (systolic blood pressure, levels of triglyceride and insulin as well as CRP, and HOMA-IR index) and regional WM or GM densities, respectively. Significant associations were observed between post-surgery improvements in plasma insulin levels and the HOMA-IR index and increased WM density in four regions: the cingulum, fornix, inferior cerebellar peduncle and posterior commissure (*P* < 0.0017 for all, Supplementary Table 2). A few significant associations were observed between post-surgery reductions in systolic blood pressure or triglyceride levels and increased WM density in the cingulum and inferior cerebellar peduncle (*P* ≤ 0.0017 for all). Increased GM density in occipital and angular regions was significantly associated with higher post-surgery systolic blood pressure (*P* < 0.0014, Supplementary Table 3). Significant associations were also observed between post-surgery improvements in plasma insulin levels and the HOMA-IR index and increased GM density in the rectus and superior motor area regions (*P* < 0.0014, Supplementary Table 3). No significant association was found between post-surgery improvements in plasma CRP levels and increased WM or GM density.

### Associations between adipocyte diameter and changes in WM and GM densities after bariatric surgery

The mean adipocyte diameter of the 29 participants from which tissue was collected at the time of surgery was 62.7 ± 8.9 μm for the OM adipose tissue and 68.9 ± 7.9 μm for the SC adipose tissue. Spearman correlations were performed to examine the associations between abdominal OM or SC adipocyte diameter and adiposity or metabolic markers. A positive and significant association was found between OM adipocyte diameter and neck circumference at baseline (0.5141, *P* < 0.0001). A positive and significant association was found between OM adipocyte diameter and insulin levels (0.3809, *P* = 0.0017), while a positive and significant association was observed between SC adipocyte diameter and triglyceride levels (0.3920, *P* = 0.0012) as well as HOMA-IR index (0.3201, *P* = 0.0093). No significant association was observed between preoperative OM or SC adipocyte diameter and the percentage of total weight loss at 24 months (*P* = 0.4184 for OM; *P* = 0.9666 for SC).

We used linear mixed-effect models (Model IV) to examine if adipocyte diameter (OM or SC) at the time of surgery was associated with post-surgery global GM and WM density changes, accounting for age and sex. The variable of interest was an interaction term between session and OM or SC, indicating whether those with higher baseline OM or SC values experienced greater changes in the VBM measurements at each session. We found a significant positive association between OM adipocyte diameter at the time of surgery and changes in total WM density at 24 months (*t* = 2.6895, *P* = 0.008), suggesting that higher OM adipocyte diameter at the time of surgery was associated with greater changes in WM density after 24 months. There was a marginally positive association between OM adipocyte diameter and total WM density after 4 months (*t* = 1.8065, *P* = 0.07) and 12 months (*t* = 1.4476, *P* = 0.15). A trend was observed between SC adipocyte diameter at the time of surgery and changes in total WM density at 24 months (*t* = 1.9748, *P* = 0.05). A marginal positive association was found between SC adipocyte diameter and total WM density at 4 months (*t* = 1.5611, *P* = 0.12). However, no significant association was observed between SC adipocyte diameter and total WM density at 12 months (*t* = 0.5517, *P* = 0.58). No significant association was found between OM or SC adipocyte diameter at the time of surgery and total GM density at 4, 12 or 24 months. The results remained similar when including diabetic status, baseline BMI and surgery type as covariates in the models.

## Discussion

In this study, we demonstrated that the increases in WM and GM densities previously observed 12 months post-surgery^22^ are still present up to 24 months post-surgery and in the same brain regions. As previously reported, these increases were more significant and extended for WM compared to GM. The increased densities, mainly in WM regions, were significantly associated with the magnitude of weight loss and metabolic improvements. We also found that larger OM adipocyte diameter at the time of surgery, which is known as a marker of adipose tissue dysfunction, was associated with greater increases in WM density 24 months after surgery. Furthermore, to ensure that the changes in WM and GM densities observed after bariatric surgery were not due to any confounding factors related to the repetition of MRI sessions and were related to weight loss induced by bariatric surgery, a separate group of participants underwent two scanning sessions before the surgery. As expected, no significant changes in GM and WM densities were observed in the two MRI sessions performed before surgery, suggesting that the neuroanatomical increases observed after the surgery are related to the weight loss and cardiometabolic improvements induced by the surgery.

To the best of our knowledge, we are the first to report that the increases in VBM WM and GM densities observed after bariatric surgery persist at 24 months. More specifically, we found widespread increases in WM density, particularly in the cerebellum, brainstem and corpus callosum, as well as less extensive increases in GM density, particularly in the cerebellum, the occipital and temporal cortex and postcentral gyrus. These results are in line with previous studies from our group^22^ and others,^24-26^ which reported mainly increases in GM and WM density over shorter follow-up periods (less than 12 months). Interestingly, our previous study indicated that these post-operative structural brain changes spatially overlapped with brain differences between individuals with severe obesity and those with normal BMI, where those with obesity typically exhibited lower VBM densities than matched controls.^22^ The observed increases in GM density are also consistent with our recent study^27^ demonstrating a significant improvement in brain health following bariatric surgery, as indicated by an impressive decrease of 2.9 and 5.6 years in brain age 12 and 24 months post-surgery, respectively. However, a recent study^32^ reported conflicting results 24 months post-surgery, showing a reduction in GM volume with no change in hippocampal and WM volume. This discrepancy may be attributed to differences in methodological approaches used to measure brain volumetry. For instance, they utilized FreeSurfer, while we and others^22,24,25^ employed the VBM method. These differences highlight the importance of considering methodological variations when interpreting changes in brain structure post-bariatric surgery.

The associations we identified between post-surgery increases in WM density and improvements in metabolic and adiposity markers are consistent with existing literature and our previous findings. Specifically, increases in WM density and resting neural activity (measured by fractional amplitude of low-frequency fluctuations) post-surgery were significantly associated with the degree of weight loss and improvements in metabolic and inflammatory profiles^22-26^. We also observed significant associations between weight loss magnitude and increases in GM density in brain regions related to cognitive processing and emotional regulation, such as the rectus, cingulate and precuneus. However, contrary associations were noted between adiposity measurements and the magnitude of total weight loss with GM density in the angular and occipital regions. Interestingly, GM density in these regions, related to sensory and cognitive perception,^44^ was positively associated with systolic blood pressure. This may help explain these findings, as no other GM regions showed similar significant associations with systolic blood pressure. Increased GM density in the superior motor area and rectus regions was associated with improvements in insulin homeostasis. Furthermore, increases in WM and GM densities were observed in the same regions at both 24 and 12 months post-surgery, with no significant difference between these sessions. This consistency could be explained by similar percentages of weight loss and metabolic improvements observed at these two time points. Taken together, these changes in brain integrity following bariatric surgery may result from weight loss and improvements in metabolic and vascular health induced by the surgery. Further investigation is needed to better understand why the associations between adiposity and metabolic variables vary with GM density across different brain regions.

The underlying mechanisms explaining the increase in WM and GM densities after bariatric surgery remain unknown. One potential mechanism is the improvement of the chronic, low-grade inflammatory state observed after bariatric surgery-induced weight loss, which can affect brain vasculature and blood–brain barrier permeability.^45-47^ In the current study, we did not find a significant association between improvements in CRP levels post-surgery and changes in GM or WM density. These results are consistent with our previous study.^22^ However, we previously reported a significant association between post-surgery reductions in lipopolysaccharide-binding protein (LBP) and increased WM density in several areas.^22^ Interestingly, a recent study reported significant reductions in several inflammatory markers 24 months post-surgery, including high-sensitivity CRP, serum amyloid A, tumour necrosis factor-α, interleukin-6 and interleukin-1β compared to baseline.^32^ They also found increased levels of adiponectin 24 months post-surgery. The same group reported significantly improved performance in all cognitive domains 6 months post-surgery, with most improvements maintained up to 24 months.^32^ This cognitive improvement after bariatric surgery was partly explained by lower CRP and leptin levels and fewer depressive symptoms.^48^ However, they did not examine the association between the improvement in inflammatory markers and brain morphological changes.

It is also possible that the structural changes identified with MRI derive from non-neuronal components of the brain, such as the vasculature, which accounts for about 5% of GM.^45^ Interestingly, a recent study found that bariatric surgery is associated with reduced brain glucose utilization, which is directly related to improvements in cognitive performance.^49^ These improvements in glucose and insulin homeostasis, reduction of inflammation and improvements in cerebral blood flow observed after bariatric surgery^50-52^ may lead to angiogenesis, neurogenesis, gliogenesis, axon sprouting and synaptic remodelling, which could influence WM and GM densities.^45,53^ An increase in WM density could also be caused by a higher number of myelinated axons in a tract or a higher thickness of myelin,^45^ although such changes would seem unlikely to happen over the time periods studied. Future studies are needed to better understand the mechanisms underlying these structural changes and their link with cognitive performance.

We also report that individuals with higher OM adipocyte diameter at the time of surgery had greater changes in total WM density 24 months post-surgery. Similar associations were observed with SC adipocyte diameter, but to a lesser extent. However, the abdominal adipocyte diameters were not related to the changes in GM density. This is consistent with the fact that the associations between the improvement of adiposity/metabolic markers and post-surgery increase in brain densities were mainly in WM. A recent review by Garcia-Garcia^18^ reported that obesity-induced inflammation is related to disruptions in WM integrity and cerebrovascular disease. Such systemic, low-grade inflammation might be the result of adipose tissue dysfunction, which is characterized by adipocyte hypertrophy.^5^ Indeed, in response to sustained positive energy imbalance, adipose tissue may reach a level at which adipocytes become dysfunctional, limiting further lipid storage in adipose tissues and leading to ectopic fat deposition and cardiometabolic alterations. As expected, we found significant associations between greater adipocyte sizes and metabolic alterations. Typically, hypertrophy is more common within OM adipose tissue than SC adipose tissue, where the expansion of adipose tissue takes place mostly through the generation of new adipocytes (adipose tissue hyperplasia).^2,3^ When adipocytes are hypertrophic, an elevated secretion of proinflammatory cytokines is observed, which can have detrimental effects on multiple organs, including the brain.^18,54^ Previous studies have found that this inflammation was reduced following bariatric surgery among many other metabolic variables.^20^ It is also important to note that WM could be more sensitive to inflammation changes observed after bariatric surgery,^24^ possibly explaining why we only observed associations between adipocyte diameter and WM density, and not GM density. Because participants with larger OM adipocyte diameter at the time of surgery had greater increases in total WM density 24 months post-surgery, this suggests that individuals with abdominal obesity might benefit the most from the bariatric surgery at the neural level.

Some limitations should be acknowledged. First, we did not include a control group of individuals with normal weight with 24 months of follow-up to assess if the changes that we found at 24 months post-surgery would be observed in a population without obesity. However, we added a separate group of participants with severe obesity who underwent two scanning sessions before surgery to ensure the effects seen after bariatric surgery were not due to any confounds or artifactual T1 signal changes related to the repetition of the MRI sessions. Even though each participant serves as their own control in this prospective study, including a control group comprising individuals with obesity who achieved weight loss through lifestyle interventions could help distinguish the effects of overall weight loss from those specific to bariatric surgery. Another limitation is that we did not have adipose tissue samples for four participants, which reduced our sample size. Even though we included the surgery type as a covariate in our statistical models, our study was not sufficiently powered to compare the different surgical interventions directly due to the small sample sizes for the two surgical groups (biliopancreatic diversion with duodenal switch and Roux-en-Y gastric bypass). However, when we conducted the analysis with only the sleeve gastrectomy group, the results remained consistent. With larger sample sizes from each surgery type, it would be interesting to explore the potential differences between restrictive and malabsorptive bariatric surgeries on brain integrity. Another limitation is that we used VBM to assess WM density, but diffusion-weighted imaging would have been a more sensitive method for evaluating changes in WM integrity.^55^

In conclusion, our results show extended increases in WM density up to 24 months post-surgery, which are related to metabolic and adiposity improvements. Greater preoperative OM adipocyte diameter is associated with a greater increase in WM density at 24 months, suggesting that individuals with higher abdominal adiposity might benefit the most from surgery. More research is needed to understand the underlying mechanisms explaining the brain structural changes after weight loss induced by bariatric surgery.

## Supplementary Material

fcae336_Supplementary_Data

## Data Availability

The data will be made available upon reasonable request. GitHub—BIC-MNI/minc-toolkit-v2: Version 2 of the minc-toolkit uses tools based on ITK version 4.x.
