# Supplementary material for: Sustained improvements in brain health and metabolic markers 24 months following bariatric surgery
Source: Brain Commun. 2024 Oct 4;6(5):fcae336. doi: 10.1093/braincomms/fcae336 (PMC11472827; doi:10.1093/braincomms/fcae336)
Supplement: fcae336_Supplementary_Data [file fcae336_supplementary_data.docx]

**Supplemental results**


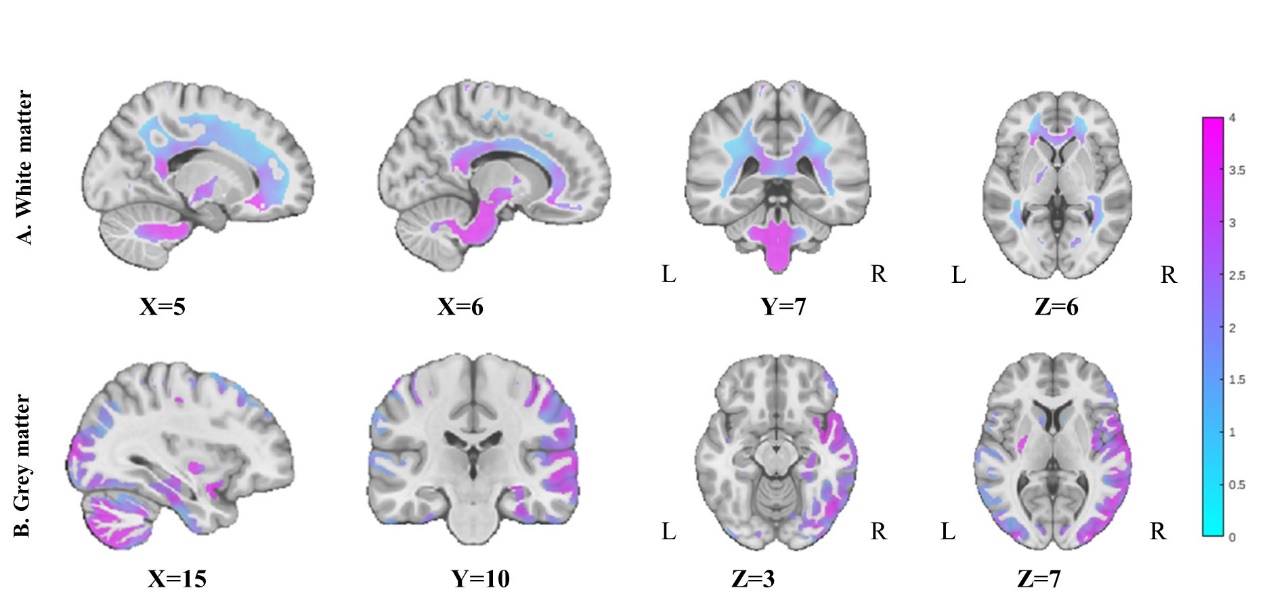


**Supplementary Figure 1:** **Changes in white matter (WM) density (A) and grey matter density (B) 24 months post-surgery compared to baseline in participants adjusted for medication intake (n=33).** The figure shows the T-value maps from the voxel-wise mixed-effects models for the WM or GM regions that were significant after whole-brain FDR correction (p < 0.05), correcting for sex, age, BMI and diabetic status at baseline, and medication intake. Colors show higher positive (in pink) or neutral (in light blue) T-values. L, left; R, right; X, Y, Z, coordinates.


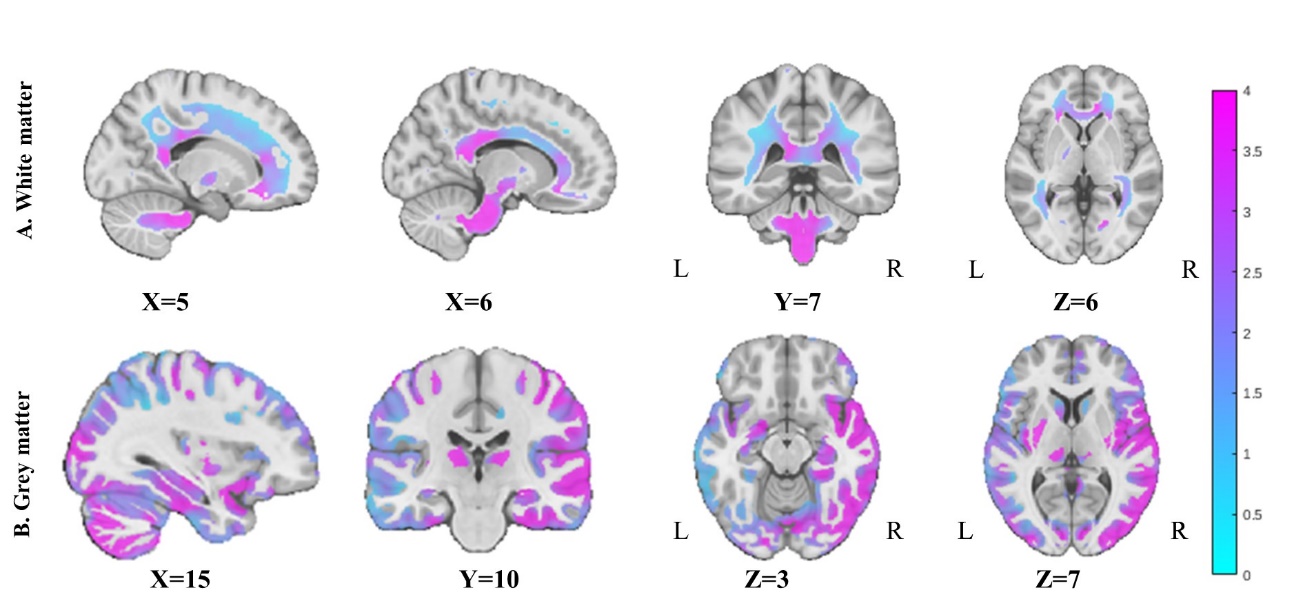


**Supplementary Figure 2:** **Changes in white matter (WM) density (A) and grey matter density (B) 24 months post-surgery compared to baseline in participants who underwent sleeve gastrectomy (N=26).** The figure shows the T-value maps from the voxel-wise mixed-effects models for the WM or GM regions that were significant after whole-brain FDR correction (p < 0.05), correcting for sex. age. as well as BMI and diabetic status at baseline. Colors show higher positive (in pink) or neutral (in light blue) T-values. L, left; R, right; X, Y, Z, coordinates.

**Supplementary Table 1**: Characteristics of the group of participants who had two pre-surgery sessions.

| **​** | **Baseline 1** | | | **Baseline 2** | | | ***p* value** |
| --- | --- | --- | --- | --- | --- | --- | --- |
| *N* | 19 | | | 19 | | |  |
| Sex (F:M)​ | 13 | :​ | 6 | 13​ | :​ | 6​ |  |
| Age (years)​ | 43.8​ | ± | 10.2 | 43.9 | ±​ | 9.0​ | 0.9884 ; t=2.02619 |
| Diabetic (Y:N)​ | 6 | ​ | 13 | 6 | : | 13 |  |
| BMI (kg/m^2^) | 41.7 | ± | 3.8 | 42.0 | ±​ | 3.6 | 0.9703; t=2.02809 |
| Waist circumference (cm) | 126.2 | ± | 10.5 | 127.1 | ± | 11.0 | 0.8000 ; t=2.02619 |
| Neck circumference (cm) | 41.7 | ± | 3.8 | 41.8 | ± | 4.3 | 0.9286 ; t=2.02619 |
| EWL (%) | - |  |  | -0.008 | ± | 0.04 | 0.3037; t=2.02809 |
| TWL (%) | - |  |  | -0.001 | ± | 0.02 | 0.4885 ; t=2.02619 |
| Systolic blood pressure (mmHg) | 128.0 | ± | 12.7 | 123.5 | ± | 12.6 | 0.2844 ; t=2.03011 |
| Diastolic blood pressure (mmHg) | 76.2 | ± | 11.9 | 74.0 | ± | 10.2 | 0.5551 ; t=2.03011 |

Results are presented as mean ± SD, F, female; M, male; Y, yes; N, no; BMI, body mass index; SG, sleeve gastrectomy; RYGB, Roux-en-Y gastric by-pass; BPD-DS, biliopancreatic derivation with duodenal switch; EWL, excess weight loss; TWL, total weight loss.

**Supplementary Table 2**: Associations between changes in white matter densities and changes in adiposity and metabolic markers in response to bariatric surgery

|  | **Name** | **BMI model** | | | | | |
| --- | --- | --- | --- | --- | --- | --- | --- |
|  |  | Tstat_BMI | Pvalue_BMI | Tstat_Age | Pvalue_Age | Tstat_Sex | Pvalue_Sex |
| 1 | Acoustic_Radiation | -3.486 | 0.001 | -0.895 | 0.373 | 1.000 | 0.319 |
| 2 | Anterior_Commissure | -1.471 | 0.144 | -1.426 | 0.156 | 0.032 | 0.974 |
| 3 | Arcuate_Fasciculus | -2.461 | 0.015 | 0.150 | 0.881 | 0.246 | 0.806 |
| 4 | Central_Tegmental_Tract (PONT) | -3.341 | 0.001 | 0.220 | 0.827 | 0.711 | 0.479 |
| 5 | Cerebellum | 0.723 | 0.471 | -0.715 | 0.476 | -0.828 | 0.409 |
| 6 | Cingulum | -6.350 | <0.0001 | -0.114 | 0.909 | 0.177 | 0.860 |
| 7 | CN | -3.578 | 0.001 | -0.042 | 0.967 | 0.678 | 0.499 |
| 8 | Corpus_Callosum and Temporopontine_Tract | -3.956 | 0.000 | -0.656 | 0.513 | 0.236 | 0.814 |
| 9 | Cortico_Spinal_Tract | -3.791 | 0.000 | -0.244 | 0.808 | 0.725 | 0.470 |
| 10 | Cortico_Striatal_Pathway | -3.765 | 0.000 | -0.668 | 0.505 | 0.184 | 0.854 |
| 11 | Corticothalamic_Pathway | -3.369 | 0.001 | -0.501 | 0.618 | 0.442 | 0.659 |
| 12 | Dorsal_Longitudinal_Fasciculus_L. Medial_Longitudinal_Fasciculus | -3.239 | 0.002 | -0.032 | 0.975 | 0.614 | 0.540 |
| 13 | Extreme_Capsule | -3.711 | 0.000 | 1.416 | 0.159 | 0.568 | 0.571 |
| 14 | Fornix | -4.421 | 0.000 | 0.469 | 0.640 | -0.554 | 0.581 |
| 15 | Frontal_Aslant_Tract | -3.058 | 0.003 | -0.529 | 0.598 | 0.936 | 0.351 |
| 16 | Frontopontine_Tract | -4.196 | <0.0001 | -0.553 | 0.581 | 0.952 | 0.343 |
| 17 | Inferior_Cerebellar_Peduncle and middle and superior | -5.565 | <0.0001 | 0.123 | 0.903 | 0.074 | 0.942 |
| 18 | Inf._Fronto_Occipital and middle and superior longitudinal fasciculus | -1.730 | 0.086 | -0.581 | 0.562 | 0.194 | 0.847 |
| 19 | Lateral_Lemniscus_and_medial_lemniscus | -4.183 | <0.0001 | -0.293 | 0.770 | 0.373 | 0.709 |
| 20 | Occipitopontine_Tract | -4.337 | <0.0001 | -0.500 | 0.618 | -0.180 | 0.858 |
| 21 | Optic_Radiation | -2.423 | 0.017 | -1.848 | 0.067 | -0.014 | 0.989 |
| 22 | Parietopontine_Tract | -4.468 | 0.000 | -0.136 | 0.892 | 0.223 | 0.824 |
| 23 | Posterior_Commissure_rubrospinal_tract_spinothalamic_tract | -4.573 | 0.000 | -0.184 | 0.854 | 0.409 | 0.684 |
| 24 | U_Fiber | -1.278 | 0.204 | -0.026 | 0.979 | 0.185 | 0.853 |
| 25 | Uncinate_Fasciculus | -2.796 | 0.006 | -0.673 | 0.502 | 0.631 | 0.529 |
| 26 | Inferior_Fronto_Occipital_Fasciculus and Vertical_Occipital_Fasciculus | -2.243 | 0.027 | -2.262 | 0.025 | -0.433 | 0.666 |
| 27 | vermis | -1.278 | 0.204 | 0.281 | 0.779 | 0.891 | 0.374 |
| **TWL model** | | | | | | | |
|  |  | Tstat_TWL | Pvalue_TWL | Tstat_ Age | Pvalue_Age | Tstat_ Sex | Pvalue_Sex |
| 1 | Acoustic_Radiation | 3.308 | 0.001 | -0.936 | 0.351 | 0.936 | 0.351 |
| 2 | Anterior_Commissure | 1.405 | 0.162 | -1.440 | 0.152 | 0.007 | 0.995 |
| 3 | Arcuate_Fasciculus | 2.383 | 0.019 | 0.130 | 0.897 | 0.226 | 0.822 |
| 4 | Central_Tegmental_Tract (PONT) | 3.614 | 0.000 | -0.054 | 0.957 | 0.555 | 0.580 |
| 5 | Cerebellum | -0.956 | 0.341 | -0.599 | 0.550 | -0.778 | 0.438 |
| 6 | Cingulum | 6.323 | <0.0001 | -0.271 | 0.787 | 0.080 | 0.936 |
| 7 | CN | 3.573 | 0.001 | -0.227 | 0.821 | 0.557 | 0.579 |
| 8 | Corpus_Callosum and Temporopontine_Tract | 3.852 | 0.000 | -0.699 | 0.486 | 0.189 | 0.850 |
| 9 | Cortico_Spinal_Tract | 3.659 | 0.000 | -0.317 | 0.752 | 0.657 | 0.512 |
| 10 | Cortico_Striatal_Pathway | 3.702 | 0.000 | -0.723 | 0.471 | 0.136 | 0.892 |
| 11 | Corticothalamic_Pathway | 3.251 | 0.002 | -0.535 | 0.593 | 0.397 | 0.692 |
| 12 | Dorsal_Longitudinal_Fasciculus_L. Medial_Longitudinal_Fasciculus | 3.514 | 0.001 | -0.295 | 0.768 | 0.467 | 0.641 |
| 13 | Extreme_Capsule | 3.622 | 0.000 | 1.351 | 0.179 | 0.532 | 0.595 |
| 14 | Fornix | 4.601 | 0.000 | 0.254 | 0.800 | -0.676 | 0.500 |
| 15 | Frontal_Aslant_Tract | 3.061 | 0.003 | -0.595 | 0.553 | 0.891 | 0.375 |
| 16 | Frontopontine_Tract | 4.127 | <0.0001 | -0.627 | 0.532 | 0.888 | 0.376 |
| 17 | Inferior_Cerebellar_Peduncle and middle and superior | 5.691 | <0.0001 | -0.126 | 0.900 | -0.072 | 0.943 |
| 18 | Inf._Fronto_Occipital and middle and superior longitudinal fasciculus | 1.594 | 0.114 | -0.555 | 0.580 | 0.188 | 0.851 |
| 19 | Lateral_Lemniscus_and_medial_lemniscus | 4.202 | <0.0001 | -0.529 | 0.598 | 0.219 | 0.827 |
| 20 | Occipitopontine_Tract | 4.179 | <0.0001 | -0.539 | 0.591 | -0.223 | 0.824 |
| 21 | Optic_Radiation | 2.322 | 0.022 | -1.867 | 0.064 | -0.052 | 0.959 |
| 22 | Parietopontine_Tract | 4.368 | 0.000 | -0.242 | 0.809 | 0.147 | 0.884 |
| 23 | Posterior_Commissure_rubrospinal_tract_spinothalamic_tract | 4.586 | 0.000 | -0.484 | 0.629 | 0.217 | 0.829 |
| 24 | U_Fiber | 1.088 | 0.279 | 0.019 | 0.985 | 0.193 | 0.848 |
| 25 | Uncinate_Fasciculus | 2.588 | 0.011 | -0.707 | 0.481 | 0.576 | 0.566 |
| 26 | Inferior_Fronto_Occipital_Fasciculus and Vertical_Occipital_Fasciculus | 2.107 | 0.037 | -2.231 | 0.027 | -0.449 | 0.654 |
| 27 | vermis | 1.217 | 0.226 | 0.255 | 0.799 | 0.870 | 0.386 |
| **Waist circumference Model** | | | | | | | |
|  |  | Tstat_waist | Pvalue_ waist | Tstat_ Age | Pvalue_Age | Tstat_Sex | Pvalue Sex |
| 1 | Acoustic_Radiation | -3.438 | 0.001 | -1.050 | 0.296 | 0.850 | 0.397 |
| 2 | Anterior_Commissure | -1.847 | 0.067 | -1.529 | 0.129 | -0.124 | 0.902 |
| 3 | Arcuate_Fasciculus | -3.208 | 0.002 | -0.145 | 0.885 | 0.095 | 0.925 |
| 4 | Central_Tegmental_Tract (PONT) | -3.445 | 0.001 | 0.419 | 0.676 | 0.829 | 0.409 |
| 5 | Cerebellum | 0.095 | 0.925 | -0.634 | 0.527 | -0.834 | 0.406 |
| 6 | Cingulum | -6.807 | <0.0001 | -0.221 | 0.826 | 0.000 | 1.000 |
| 7 | CN | -3.713 | 0.000 | 0.046 | 0.963 | 0.601 | 0.549 |
| 8 | Corpus_Callosum and Temporopontine_Tract | -4.394 | 0.000 | -0.827 | 0.410 | 0.059 | 0.953 |
| 9 | Cortico_Spinal_Tract | -3.882 | 0.000 | -0.387 | 0.699 | 0.538 | 0.592 |
| 10 | Cortico_Striatal_Pathway | -4.266 | 0.000 | -0.923 | 0.358 | -0.026 | 0.979 |
| 11 | Corticothalamic_Pathway | -3.823 | 0.000 | -0.717 | 0.475 | 0.237 | 0.813 |
| 12 | Dorsal_Longitudinal_Fasciculus_L. Medial_Longitudinal_Fasciculus | -3.371 | 0.001 | 0.173 | 0.863 | 0.719 | 0.474 |
| 13 | Extreme_Capsule | -3.831 | 0.000 | 1.134 | 0.259 | 0.402 | 0.688 |
| 14 | Fornix | -4.272 | 0.000 | 0.454 | 0.651 | -0.669 | 0.505 |
| 15 | Frontal_Aslant_Tract | -3.720 | 0.000 | -0.746 | 0.458 | 0.789 | 0.432 |
| 16 | Frontopontine_Tract | -4.508 | <0.0001 | -0.712 | 0.478 | 0.791 | 0.431 |
| 17 | Inferior_Cerebellar_Peduncle and middle and superior | -5.624 | <0.0001 | 0.163 | 0.871 | -0.055 | 0.956 |
| 18 | Inf._Fronto_Occipital and middle and superior longitudinal fasciculus | -2.220 | 0.028 | -0.631 | 0.529 | 0.117 | 0.907 |
| 19 | Lateral_Lemniscus_and_medial_lemniscus | -4.075 | <0.0001 | -0.180 | 0.858 | 0.330 | 0.742 |
| 20 | Occipitopontine_Tract | -4.412 | <0.0001 | -0.494 | 0.622 | -0.277 | 0.782 |
| 21 | Optic_Radiation | -2.626 | 0.010 | -1.907 | 0.059 | -0.179 | 0.859 |
| 22 | Parietopontine_Tract | -4.515 | 0.000 | -0.345 | 0.731 | -0.009 | 0.993 |
| 23 | Posterior_Commissure_rubrospinal_tract_spinothalamic_tract | -4.493 | 0.000 | -0.021 | 0.983 | 0.310 | 0.757 |
| 24 | U_Fiber | -1.718 | 0.089 | -0.134 | 0.894 | 0.089 | 0.929 |
| 25 | Uncinate_Fasciculus | -2.763 | 0.007 | -0.762 | 0.448 | 0.556 | 0.579 |
| 26 | Inferior_Fronto_Occipital_Fasciculus and Vertical_Occipital_Fasciculus | -2.746 | 0.007 | -2.339 | 0.021 | -0.576 | 0.566 |
| 27 | vermis | -1.785 | 0.077 | 0.220 | 0.826 | 0.790 | 0.431 |
| **Neck circumference model** | | | | | | | |
|  |  | Tstat_neck_cir | Pvalue_neck_cir | Tstat_Age | Pvalue_Age | Tstat_Sex | Pvalue_Sex |
| 1 | Acoustic_Radiation | -3.087 | 0.003 | -1.185 | 0.239 | 0.015 | 0.988 |
| 2 | Anterior_Commissure | -1.146 | 0.255 | -1.586 | 0.116 | -0.366 | 0.715 |
| 3 | Arcuate_Fasciculus | -2.000 | 0.048 | -0.147 | 0.883 | -0.174 | 0.862 |
| 4 | Central_Tegmental_Tract (PONT) | -2.490 | 0.015 | -0.581 | 0.562 | -0.922 | 0.359 |
| 5 | Cerebellum | 0.466 | 0.642 | -0.786 | 0.434 | -0.828 | 0.410 |
| 6 | Cingulum | -5.129 | <0.0001 | -0.334 | 0.739 | -0.767 | 0.445 |
| 7 | CN | -3.249 | 0.002 | -0.722 | 0.472 | -0.898 | 0.371 |
| 8 | Corpus_Callosum and Temporopontine_Tract | -3.110 | 0.003 | -0.768 | 0.444 | -0.357 | 0.722 |
| 9 | Cortico_Spinal_Tract | -3.293 | 0.001 | -0.422 | 0.674 | -0.162 | 0.871 |
| 10 | Cortico_Striatal_Pathway | -3.035 | 0.003 | -0.773 | 0.441 | -0.365 | 0.716 |
| 11 | Corticothalamic_Pathway | -2.704 | 0.008 | -0.655 | 0.514 | -0.180 | 0.857 |
| 12 | Dorsal_Longitudinal_Fasciculus_L. Medial_Longitudinal_Fasciculus | -2.470 | 0.015 | -0.720 | 0.473 | -0.899 | 0.371 |
| 13 | Extreme_Capsule | -3.123 | 0.002 | 1.275 | 0.206 | 0.113 | 0.911 |
| 14 | Fornix | -3.034 | 0.003 | 0.136 | 0.892 | -1.134 | 0.260 |
| 15 | Frontal_Aslant_Tract | -2.515 | 0.014 | -0.663 | 0.509 | 0.484 | 0.630 |
| 16 | Frontopontine_Tract | -3.661 | 0.000 | -0.730 | 0.467 | 0.189 | 0.850 |
| 17 | Inferior_Cerebellar_Peduncle and middle and superior | -4.826 | <0.0001 | -0.414 | 0.680 | -1.455 | 0.149 |
| 18 | Inf._Fronto_Occipital and middle and superior longitudinal fasciculus | -1.170 | 0.245 | -0.714 | 0.477 | -0.082 | 0.935 |
| 19 | Lateral_Lemniscus_and_medial_lemniscus | -3.444 | 0.001 | -0.980 | 0.330 | -1.386 | 0.169 |
| 20 | Occipitopontine_Tract | -3.347 | 0.001 | -0.627 | 0.532 | -0.875 | 0.384 |
| 21 | Optic_Radiation | -2.088 | 0.040 | -2.100 | 0.038 | -0.615 | 0.540 |
| 22 | Parietopontine_Tract | -3.769 | 0.000 | -0.201 | 0.841 | -0.594 | 0.554 |
| 23 | Posterior_Commissure_rubrospinal_tract_spinothalamic_tract | -3.658 | 0.000 | -0.948 | 0.346 | -1.610 | 0.111 |
| 24 | U_Fiber | -0.695 | 0.489 | -0.135 | 0.893 | 0.018 | 0.986 |
| 25 | Uncinate_Fasciculus | -2.015 | 0.047 | -0.864 | 0.390 | -0.106 | 0.916 |
| 26 | Inferior_Fronto_Occipital_Fasciculus and Vertical_Occipital_Fasciculus | -1.688 | 0.095 | -2.345 | 0.021 | -0.833 | 0.407 |
| 27 | Vermis | -1.496 | 0.138 | 0.015 | 0.988 | 0.415 | 0.679 |
| **SBP model** | | | | | | | |
|  |  | Tstat_SBP | Pvalue_ SBP | Tstat Age | Pvalue_Age | Tstat_Sex | Pvalue_Sex |
| 1 | Acoustic_Radiation | -2.390 | 0.018 | -0.276 | 0.783 | 1.077 | 0.284 |
| 2 | Anterior_Commissure | -0.775 | 0.440 | -1.148 | 0.253 | 0.102 | 0.919 |
| 3 | Arcuate_Fasciculus | -1.129 | 0.261 | 1.143 | 0.255 | 0.529 | 0.597 |
| 4 | Central_Tegmental_Tract (PONT) | -1.748 | 0.083 | 0.437 | 0.663 | 0.583 | 0.561 |
| 5 | Cerebellum | 1.091 | 0.277 | -0.740 | 0.461 | -0.773 | 0.441 |
| 6 | Cingulum | -3.847 | 0.000 | 1.603 | 0.111 | 0.619 | 0.537 |
| 7 | CN | -2.436 | 0.016 | 0.330 | 0.742 | 0.606 | 0.546 |
| 8 | Corpus_Callosum and Temporopontine_Tract | -2.294 | 0.023 | 0.494 | 0.622 | 0.545 | 0.587 |
| 9 | Cortico_Spinal_Tract | -2.183 | 0.031 | 0.567 | 0.572 | 0.889 | 0.376 |
| 10 | Cortico_Striatal_Pathway | -2.154 | 0.033 | 0.483 | 0.630 | 0.502 | 0.616 |
| 11 | Corticothalamic_Pathway | -1.892 | 0.061 | 0.403 | 0.687 | 0.672 | 0.503 |
| 12 | Dorsal_Longitudinal_Fasciculus_L. Medial_Longitudinal_Fasciculus | -1.644 | 0.103 | 0.197 | 0.844 | 0.512 | 0.609 |
| 13 | Extreme_Capsule | -2.144 | 0.034 | 2.864 | 0.005 | 0.926 | 0.356 |
| 14 | Fornix | -2.775 | 0.006 | 1.494 | 0.138 | -0.305 | 0.761 |
| 15 | Frontal_Aslant_Tract | -1.502 | 0.136 | 0.682 | 0.496 | 1.268 | 0.207 |
| 16 | Frontopontine_Tract | -2.536 | 0.012 | 0.591 | 0.555 | 1.224 | 0.223 |
| 17 | Inferior_Cerebellar_Peduncle and middle and superior | -3.624 | 0.000 | 0.979 | 0.329 | 0.194 | 0.847 |
| 18 | Inf._Fronto_Occipital and middle and superior longitudinal fasciculus | -0.627 | 0.532 | 0.037 | 0.971 | 0.389 | 0.698 |
| 19 | Lateral_Lemniscus_and_medial_lemniscus | -2.649 | 0.009 | 0.096 | 0.924 | 0.272 | 0.786 |
| 20 | Occipitopontine_Tract | -2.699 | 0.008 | 0.581 | 0.562 | 0.107 | 0.915 |
| 21 | Optic_Radiation | -1.788 | 0.076 | -1.448 | 0.150 | 0.053 | 0.958 |
| 22 | Parietopontine_Tract | -2.676 | 0.008 | 0.818 | 0.415 | 0.438 | 0.662 |
| 23 | Posterior_Commissure_rubrospinal_tract_spinothalamic_tract | -2.855 | 0.005 | 0.134 | 0.893 | 0.222 | 0.825 |
| 24 | U_Fiber | -0.403 | 0.688 | 0.431 | 0.667 | 0.325 | 0.746 |
| 25 | Uncinate_Fasciculus | -1.602 | 0.112 | -0.187 | 0.852 | 0.692 | 0.490 |
| 26 | Inferior_Fronto_Occipital_Fasciculus and Vertical_Occipital_Fasciculus | -1.084 | 0.280 | -1.684 | 0.095 | -0.246 | 0.806 |
| 27 | vermis | -0.699 | 0.486 | 0.557 | 0.578 | 0.953 | 0.343 |
| **TG model** | | | | | | | |
|  |  | Tstat_ TG | Pvalue_ TG | Tstat_Age | PvalueAge | Tstat_Sex | Pvalue_Sex |
| 1 | Acoustic_Radiation | -2.276 | 0.025 | -0.301 | 0.764 | 1.227 | 0.222 |
| 2 | Anterior_Commissure | -2.143 | 0.034 | -1.388 | 0.168 | 0.161 | 0.872 |
| 3 | Arcuate_Fasciculus | -2.231 | 0.028 | 0.861 | 0.391 | 0.525 | 0.601 |
| 4 | Central_Tegmental_Tract (PONT) | -2.480 | 0.015 | 0.587 | 0.559 | 0.883 | 0.379 |
| 5 | Cerebellum | 0.300 | 0.765 | -0.885 | 0.378 | -0.804 | 0.423 |
| 6 | Cingulum | -3.499 | 0.001 | 1.598 | 0.113 | 0.753 | 0.453 |
| 7 | CN | -2.166 | 0.032 | 0.477 | 0.634 | 0.871 | 0.386 |
| 8 | Corpus_Callosum and Temporopontine_Tract | -2.631 | 0.010 | 0.412 | 0.681 | 0.651 | 0.517 |
| 9 | Cortico_Spinal_Tract | -2.352 | 0.020 | 0.572 | 0.568 | 1.051 | 0.296 |
| 10 | Cortico_Striatal_Pathway | -2.440 | 0.016 | 0.416 | 0.678 | 0.602 | 0.548 |
| 11 | Corticothalamic_Pathway | -2.417 | 0.017 | 0.263 | 0.793 | 0.758 | 0.450 |
| 12 | Dorsal_Longitudinal_Fasciculus_L. Medial_Longitudinal_Fasciculus | -2.375 | 0.019 | 0.329 | 0.743 | 0.844 | 0.401 |
| 13 | Extreme_Capsule | -2.418 | 0.017 | 2.875 | 0.005 | 1.018 | 0.311 |
| 14 | Fornix | -3.156 | 0.002 | 1.407 | 0.162 | -0.161 | 0.872 |
| 15 | Frontal_Aslant_Tract | -1.877 | 0.063 | 0.633 | 0.528 | 1.338 | 0.183 |
| 16 | Frontopontine_Tract | -2.250 | 0.026 | 0.678 | 0.499 | 1.388 | 0.168 |
| 17 | Inferior_Cerebellar_Peduncle and middle and superior | -3.378 | 0.001 | 1.072 | 0.286 | 0.418 | 0.677 |
| 18 | Inf._Fronto_Occipital and middle and superior longitudinal fasciculus | -1.284 | 0.202 | -0.091 | 0.928 | 0.452 | 0.652 |
| 19 | Lateral_Lemniscus_and_medial_lemniscus | -2.653 | 0.009 | 0.185 | 0.853 | 0.523 | 0.602 |
| 20 | Occipitopontine_Tract | -2.793 | 0.006 | 0.570 | 0.570 | 0.241 | 0.810 |
| 21 | Optic_Radiation | -2.177 | 0.032 | -1.536 | 0.127 | 0.228 | 0.820 |
| 22 | Parietopontine_Tract | -2.763 | 0.007 | 0.827 | 0.410 | 0.615 | 0.540 |
| 23 | Posterior_Commissure_rubrospinal_tract_spinothalamic_tract | -2.408 | 0.018 | 0.231 | 0.818 | 0.597 | 0.552 |
| 24 | U_Fiber | -1.115 | 0.267 | 0.359 | 0.721 | 0.418 | 0.677 |
| 25 | Uncinate_Fasciculus | -2.347 | 0.021 | -0.207 | 0.836 | 0.851 | 0.397 |
| 26 | Inferior_Fronto_Occipital_Fasciculus and Vertical_Occipital_Fasciculus | -2.165 | 0.032 | -1.986 | 0.049 | -0.213 | 0.832 |
| 27 | vermis | -1.125 | 0.263 | 0.511 | 0.611 | 1.070 |  |
| **Insulin model** | | | | | | | |
|  |  | Tstat_ Insulin | Pvalue_ Insulin | Tstat_ _Age | Pvalue_Age | Tstat_Sex | Pvalue_Sex |
| 1 | Acoustic_Radiation | -2.533 | 0.013 | -0.575 | 0.566 | 1.046 | 0.298 |
| 2 | Anterior_Commissure | -1.846 | 0.068 | -1.291 | 0.199 | 0.111 | 0.912 |
| 3 | Arcuate_Fasciculus | -2.030 | 0.045 | 0.624 | 0.534 | 0.432 | 0.667 |
| 4 | Central_Tegmental_Tract (PONT) | -2.439 | 0.016 | 0.435 | 0.665 | 0.555 | 0.580 |
| 5 | Cerebellum | -0.197 | 0.844 | -0.746 | 0.457 | -0.657 | 0.513 |
| 6 | Cingulum | -3.608 | 0.001 | 0.904 | 0.368 | 0.419 | 0.676 |
| 7 | CN | -2.624 | 0.010 | 0.258 | 0.797 | 0.673 | 0.503 |
| 8 | Corpus_Callosum and Temporopontine_Tract | -2.812 | 0.006 | 0.008 | 0.994 | 0.438 | 0.663 |
| 9 | Cortico_Spinal_Tract | -2.881 | 0.005 | 0.183 | 0.856 | 0.825 | 0.411 |
| 10 | Cortico_Striatal_Pathway | -2.868 | 0.005 | -0.047 | 0.963 | 0.380 | 0.705 |
| 11 | Corticothalamic_Pathway | -2.427 | 0.017 | -0.007 | 0.995 | 0.591 | 0.556 |
| 12 | Dorsal_Longitudinal_Fasciculus_L. Medial_Longitudinal_Fasciculus | -2.450 | 0.016 | 0.193 | 0.848 | 0.541 | 0.590 |
| 13 | Extreme_Capsule | -2.620 | 0.010 | 2.331 | 0.022 | 0.829 | 0.409 |
| 14 | Fornix | -3.818 | 0.000 | 0.828 | 0.410 | -0.496 | 0.621 |
| 15 | Frontal_Aslant_Tract | -2.414 | 0.017 | 0.150 | 0.881 | 1.164 | 0.247 |
| 16 | Frontopontine_Tract | -3.007 | 0.003 | 0.116 | 0.908 | 1.138 | 0.257 |
| 17 | Inferior_Cerebellar_Peduncle and middle and superior | -3.963 | 0.000 | 0.653 | 0.515 | 0.107 | 0.915 |
| 18 | Inf._Fronto_Occipital and middle and superior longitudinal fasciculus | -1.593 | 0.114 | -0.244 | 0.808 | 0.370 | 0.712 |
| 19 | Lateral_Lemniscus_and_medial_lemniscus | -2.999 | 0.003 | -0.070 | 0.945 | 0.218 | 0.828 |
| 20 | Occipitopontine_Tract | -2.873 | 0.005 | 0.213 | 0.832 | 0.028 | 0.978 |
| 21 | Optic_Radiation | -1.812 | 0.073 | -1.482 | 0.141 | 0.127 | 0.899 |
| 22 | Parietopontine_Tract | -3.127 | 0.002 | 0.408 | 0.684 | 0.348 | 0.729 |
| 23 | Posterior_Commissure_rubrospinal_tract_spinothalamic_tract | -3.625 | 0.000 | -0.021 | 0.983 | 0.239 | 0.812 |
| 24 | U_Fiber | -1.215 | 0.227 | 0.358 | 0.721 | 0.419 | 0.676 |
| 25 | Uncinate_Fasciculus | -2.826 | 0.006 | -0.387 | 0.700 | 0.719 | 0.474 |
| 26 | Inferior_Fronto_Occipital_Fasciculus and Vertical_Occipital_Fasciculus | -2.027 | 0.045 | -1.999 | 0.048 | -0.317 | 0.752 |
| 27 | vermis | -1.223 | 0.224 | 0.511 | 0.610 | 1.083 | 0.281 |
| **HOMA-IR model** | | | | | | | |
|  |  | Tstat_  HOMAIR | Pvalue_  HOMAIR | Tstat_Age | PvalueAge | Tstat_ Sex | Pvalue_ Sex |
| 1 | Acoustic_Radiation | -2.392 | 0.018 | -0.522 | 0.603 | 1.080 | 0.283 |
| 2 | Anterior_Commissure | -1.842 | 0.068 | -1.272 | 0.206 | 0.128 | 0.898 |
| 3 | Arcuate_Fasciculus | -2.011 | 0.047 | 0.668 | 0.505 | 0.451 | 0.653 |
| 4 | Central_Tegmental_Tract (PONT) | -2.333 | 0.021 | 0.468 | 0.641 | 0.594 | 0.553 |
| 5 | Cerebellum | -0.470 | 0.639 | -0.799 | 0.426 | -0.681 | 0.497 |
| 6 | Cingulum | -3.573 | 0.001 | 0.984 | 0.327 | 0.458 | 0.648 |
| 7 | CN | -2.496 | 0.014 | 0.301 | 0.764 | 0.708 | 0.481 |
| 8 | Corpus_Callosum and Temporopontine_Tract | -2.743 | 0.007 | 0.073 | 0.942 | 0.470 | 0.639 |
| 9 | Cortico_Spinal_Tract | -2.638 | 0.010 | 0.266 | 0.791 | 0.870 | 0.387 |
| 10 | Cortico_Striatal_Pathway | -2.790 | 0.006 | 0.024 | 0.981 | 0.414 | 0.680 |
| 11 | Corticothalamic_Pathway | -2.378 | 0.019 | 0.042 | 0.967 | 0.617 | 0.538 |
| 12 | Dorsal_Longitudinal_Fasciculus_L. Medial_Longitudinal_Fasciculus | -2.392 | 0.018 | 0.223 | 0.824 | 0.578 | 0.565 |
| 13 | Extreme_Capsule | -2.509 | 0.014 | 2.424 | 0.017 | 0.864 | 0.389 |
| 14 | Fornix | -3.797 | 0.000 | 0.885 | 0.378 | -0.462 | 0.645 |
| 15 | Frontal_Aslant_Tract | -2.331 | 0.022 | 0.226 | 0.822 | 1.194 | 0.235 |
| 16 | Frontopontine_Tract | -2.880 | 0.005 | 0.202 | 0.841 | 1.175 | 0.243 |
| 17 | Inferior_Cerebellar_Peduncle and middle and superior | -3.780 | 0.000 | 0.724 | 0.471 | 0.157 | 0.875 |
| 18 | Inf._Fronto_Occipital and middle and superior longitudinal fasciculus | -1.468 | 0.145 | -0.187 | 0.852 | 0.398 | 0.691 |
| 19 | Lateral_Lemniscus_and_medial_lemniscus | -2.926 | 0.004 | -0.030 | 0.976 | 0.259 | 0.796 |
| 20 | Occipitopontine_Tract | -2.722 | 0.008 | 0.288 | 0.774 | 0.067 | 0.947 |
| 21 | Optic_Radiation | -1.724 | 0.087 | -1.449 | 0.150 | 0.150 | 0.881 |
| 22 | Parietopontine_Tract | -2.913 | 0.004 | 0.489 | 0.626 | 0.393 | 0.695 |
| 23 | Posterior_Commissure_rubrospinal_tract_spinothalamic_tract | -3.533 | 0.001 | 0.026 | 0.979 | 0.293 | 0.770 |
| 24 | U_Fiber | -1.122 | 0.264 | 0.400 | 0.690 | 0.440 | 0.661 |
| 25 | Uncinate_Fasciculus | -2.733 | 0.007 | -0.333 | 0.739 | 0.749 | 0.455 |
| 26 | Inferior_Fronto_Occipital_Fasciculus and Vertical_Occipital_Fasciculus | -2.008 | 0.047 | -1.972 | 0.051 | -0.297 | 0.767 |
| 27 | vermis | -1.507 | 0.135 | 0.466 | 0.642 | 1.067 | 0.288 |
| **CRP Model** | | | | | | | |
|  |  | Tstat_  CRP | Pvalue_  CRP | Tstat_Age | PvalueAge | Tstat_ Sex | Pvalue_ Sex |
| 1 | Acoustic_Radiation | -1.320 | 0.193 | -1.980 | 0.053 | -1.510 | 0.136 |
| 2 | Anterior_Commissure | 0.000 | 0.999 | -3.500 | 0.001 | -0.240 | 0.809 |
| 3 | Arcuate_Fasciculus | -1.170 | 0.246 | -1.300 | 0.198 | -0.320 | 0.748 |
| 4 | Central_Tegmental_Tract (PONT) | -0.650 | 0.522 | -0.210 | 0.833 | -1.190 | 0.240 |
| 5 | Cerebellum | 0.780 | 0.439 | -1.960 | 0.055 | 1.650 | 0.105 |
| 6 | Cingulum | -1.470 | 0.148 | -3.830 | 0.0003 | 1.280 | 0.206 |
| 7 | CN | -1.080 | 0.284 | -0.500 | 0.619 | -0.740 | 0.460 |
| 8 | Corpus_Callosum and Temporopontine_Tract | -1.210 | 0.231 | -3.230 | 0.002 | 0.030 | 0.974 |
| 9 | Cortico_Spinal_Tract | -1.630 | 0.110 | -1.090 | 0.280 | -1.040 | 0.303 |
| 10 | Cortico_Striatal_Pathway | -1.040 | 0.303 | -3.240 | 0.002 | -0.140 | 0.892 |
| 11 | Corticothalamic_Pathway | -1.230 | 0.225 | -2.820 | 0.007 | -0.480 | 0.631 |
| 12 | Dorsal_Longitudinal_Fasciculus_L. Medial_Longitudinal_Fasciculus | -0.510 | 0.613 | -0.440 | 0.661 | -1.060 | 0.293 |
| 13 | Extreme_Capsule | -1.530 | 0.133 | -0.160 | 0.873 | -0.620 | 0.537 |
| 14 | Fornix | -0.100 | 0.921 | 0.120 | 0.904 | 1.060 | 0.296 |
| 15 | Frontal_Aslant_Tract | -1.530 | 0.132 | -2.710 | 0.010 | -0.980 | 0.330 |
| 16 | Frontopontine_Tract | -1.370 | 0.176 | -3.190 | 0.002 | -0.820 | 0.417 |
| 17 | Inferior_Cerebellar_Peduncle and middle and superior | -0.210 | 0.831 | -0.700 | 0.489 | 0.200 | 0.844 |
| 18 | Inf._Fronto_Occipital and middle and superior longitudinal fasciculus | -0.900 | 0.371 | -2.580 | 0.013 | -0.580 | 0.563 |
| 19 | Lateral_Lemniscus_and_medial_lemniscus | -0.280 | 0.780 | -0.540 | 0.590 | -0.740 | 0.460 |
| 20 | Occipitopontine_Tract | -0.210 | 0.832 | -2.620 | 0.011 | 0.630 | 0.530 |
| 21 | Optic_Radiation | 0.040 | 0.971 | -3.320 | 0.002 | 0.070 | 0.942 |
| 22 | Parietopontine_Tract | -1.890 | 0.064 | -1.300 | 0.201 | -0.380 | 0.708 |
| 23 | Posterior_Commissure_rubrospinal_tract_spinothalamic_tract | -0.270 | 0.788 | -0.270 | 0.786 | -0.880 | 0.382 |
| 24 | U_Fiber | -1.030 | 0.306 | -1.890 | 0.064 | -0.560 | 0.577 |
| 25 | Uncinate_Fasciculus | -1.000 | 0.323 | -2.760 | 0.008 | -1.630 | 0.109 |
| 26 | Inferior_Fronto_Occipital_Fasciculus and Vertical_Occipital_Fasciculus | -0.300 | 0.766 | -4.630 | 0.0001 | 0.250 | 0.800 |
| 27 | vermis | -0.570 | 0.572 | -0.970 | 0.337 | -0.120 | 0.904 |

Results are presented as T-stat and p-value. Model used: Adjusted VBM changes ∼ changes in adiposity/metabolic/inflammatory variables + Age + Sex. BMI, body mass index; TWL, total weight loss; SBP, systolic blood pressure; TG, triglycerides levels; HOMA-IR, homeostasis model assessment of insulin resistance; CRP, C-Reactive Protein

**Supplementary Table 3:** Associations between changes in grey matter densities and changes adiposity and metabolic markers in response to the bariatric surgery

| **BMI model** | | | | | | | |
| --- | --- | --- | --- | --- | --- | --- | --- |
|  |  | Tstat_ BMI | Pvalue_ BMI | Tstat Age | Pvalue_ Age | Tstat_ Sex | Pvalue_ Sex |
| 1 | precentral | 1.656 | 0.100 | -1.010 | 0.314 | -1.978 | 0.050 |
| 2 | frontal sup | -0.034 | 0.973 | -1.394 | 0.166 | -2.236 | 0.027 |
| 3 | frontal mid | 1.906 | 0.059 | -1.249 | 0.214 | -3.268 | 0.001 |
| 4 | frontal inf oper | 1.710 | 0.090 | -1.372 | 0.172 | -1.574 | 0.118 |
| 5 | frontal inf tri and mid | 1.126 | 0.262 | -3.294 | 0.001 | -2.095 | 0.038 |
| 6 | rolandic oper | 0.912 | 0.364 | -0.882 | 0.379 | -1.200 | 0.232 |
| 7 | sup motor area | -2.807 | 0.006 | -0.433 | 0.666 | -1.009 | 0.315 |
| 8 | olfactory | -2.048 | 0.043 | 1.059 | 0.292 | 0.258 | 0.797 |
| 9 | frontal sup medial | -1.491 | 0.139 | -1.644 | 0.103 | -1.632 | 0.105 |
| 10 | frontal med orb | -2.169 | 0.032 | -1.112 | 0.268 | -1.497 | 0.137 |
| 11 | rectus | -4.130 | 0.000 | -1.213 | 0.227 | 0.251 | 0.802 |
| 12 | OFC | -2.229 | 0.028 | -0.817 | 0.415 | -0.812 | 0.418 |
| 13 | insula | -0.120 | 0.905 | -0.793 | 0.429 | 0.613 | 0.541 |
| 14 | cingulate | -4.005 | 0.000 | -0.165 | 0.869 | -1.242 | 0.216 |
| 15 | hippocampus | -1.055 | 0.294 | -0.147 | 0.883 | -0.411 | 0.682 |
| 16 | parahipoccampal | -1.023 | 0.308 | 0.679 | 0.498 | -1.143 | 0.255 |
| 17 | amygdala | 0.765 | 0.446 | 1.806 | 0.073 | 0.212 | 0.832 |
| 18 | calcarine | -2.191 | 0.030 | 0.001 | 0.999 | -2.369 | 0.019 |
| 19 | cuneus | -1.252 | 0.213 | -0.142 | 0.888 | -1.548 | 0.124 |
| 20 | lingual | -2.377 | 0.019 | -0.014 | 0.989 | -1.167 | 0.245 |
| 21 | occipital | 3.510 | 0.001 | -1.138 | 0.257 | -1.417 | 0.159 |
| 22 | fusiform | -0.880 | 0.381 | -0.712 | 0.478 | -1.214 | 0.227 |
| 23 | postcentral | 1.798 | 0.074 | -0.363 | 0.717 | -0.851 | 0.396 |
| 24 | parietal and supramarginal | 1.361 | 0.176 | -0.222 | 0.824 | -2.241 | 0.027 |
| 25 | angular | 4.194 | 0.000 | -0.942 | 0.348 | -0.533 | 0.595 |
| 26 | precuneus | -4.399 | 0.000 | -0.267 | 0.790 | -2.364 | 0.020 |
| 27 | paracentral lobule | -3.554 | 0.001 | 0.206 | 0.837 | -1.814 | 0.072 |
| 28 | caudate | -1.742 | 0.084 | 0.383 | 0.702 | 0.489 | 0.626 |
| 29 | putamen | 0.213 | 0.832 | 0.552 | 0.582 | 0.207 | 0.837 |
| 30 | pallidum and thalamus | -0.417 | 0.677 | -0.569 | 0.570 | -1.554 | 0.123 |
| 31 | hesch | 1.028 | 0.306 | -0.303 | 0.763 | -0.215 | 0.830 |
| 32 | temporal | 2.089 | 0.039 | 0.205 | 0.838 | -0.083 | 0.934 |
| 33 | cerebellum | 0.515 | 0.608 | 0.256 | 0.799 | -1.558 | 0.122 |
| 34 | Vermis | -2.793 | 0.006 | 1.407 | 0.162 | -2.694 | 0.008 |
| **TWL model** | | | | | | | |
|  |  | Tstat_ TWL | Pvalue_ TWL | Tstat_ Age | Pvalue _Age | Tstat_ Sex | Pvalue_ Sex |
| 1 | precentral | -2.175 | 0.031 | -0.800 | 0.425 | -1.918 | 0.057 |
| 2 | frontal sup | -0.210 | 0.834 | -1.302 | 0.195 | -2.202 | 0.029 |
| 3 | frontal mid | -2.126 | 0.035 | -1.095 | 0.276 | -3.231 | 0.002 |
| 4 | frontal inf oper | -1.798 | 0.074 | -1.267 | 0.207 | -1.530 | 0.128 |
| 5 | frontal inf tri and mid | -1.116 | 0.267 | -3.217 | 0.002 | -2.066 | 0.041 |
| 6 | rolandic oper | -1.208 | 0.229 | -0.756 | 0.451 | -1.148 | 0.253 |
| 7 | sup motor area | 2.521 | 0.013 | -0.465 | 0.642 | -1.029 | 0.305 |
| 8 | olfactory | 2.154 | 0.033 | 0.908 | 0.366 | 0.172 | 0.863 |
| 9 | frontal sup medial | 1.267 | 0.208 | -1.593 | 0.114 | -1.616 | 0.108 |
| 10 | frontal med orb | 1.958 | 0.052 | -1.119 | 0.265 | -1.505 | 0.135 |
| 11 | rectus | 4.218 | 0.000 | -1.429 | 0.155 | 0.102 | 0.919 |
| 12 | OFC | 2.176 | 0.031 | -0.900 | 0.370 | -0.869 | 0.386 |
| 13 | insula | 0.000 | 1.000 | -0.781 | 0.436 | 0.613 | 0.541 |
| 14 | cingulate | 3.935 | 0.000 | -0.339 | 0.735 | -1.337 | 0.183 |
| 15 | hippocampus | 0.934 | 0.352 | -0.198 | 0.843 | -0.448 | 0.655 |
| 16 | parahipoccampal | 1.049 | 0.296 | 0.611 | 0.542 | -1.177 | 0.241 |
| 17 | amygdala | -0.698 | 0.486 | 1.811 | 0.072 | 0.233 | 0.816 |
| 18 | calcarine | 2.075 | 0.040 | -0.063 | 0.949 | -2.394 | 0.018 |
| 19 | cuneus | 1.217 | 0.226 | -0.179 | 0.858 | -1.569 | 0.119 |
| 20 | lingual | 2.413 | 0.017 | -0.124 | 0.902 | -1.231 | 0.221 |
| 21 | occipital | -3.569 | 0.001 | -0.940 | 0.349 | -1.310 | 0.192 |
| 22 | fusiform | 0.947 | 0.345 | -0.769 | 0.443 | -1.248 | 0.214 |
| 23 | postcentral | -1.936 | 0.055 | -0.237 | 0.813 | -0.789 | 0.432 |
| 24 | parietal and supramarginal | -1.905 | 0.059 | -0.005 | 0.996 | -2.191 | 0.030 |
| 25 | angular | -4.554 | 0.000 | -0.650 | 0.517 | -0.380 | 0.705 |
| 26 | precuneus | 4.083 | 0.000 | -0.353 | 0.725 | -2.342 | 0.021 |
| 27 | paracentral lobule | 3.457 | 0.001 | 0.084 | 0.933 | -1.870 | 0.064 |
| 28 | caudate | 1.703 | 0.091 | 0.272 | 0.786 | 0.418 | 0.677 |
| 29 | putamen | -0.747 | 0.457 | 0.632 | 0.528 | 0.251 | 0.803 |
| 30 | pallidum and thalamus | 0.217 | 0.828 | -0.576 | 0.565 | -1.564 | 0.120 |
| 31 | hesch | -1.089 | 0.278 | -0.233 | 0.816 | -0.176 | 0.860 |
| 32 | temporal | -2.188 | 0.030 | 0.348 | 0.728 | 0.002 | 0.998 |
| 33 | cerebellum | -0.186 | 0.853 | 0.244 | 0.807 | -1.539 | 0.126 |
| 34 | vermis | 3.604 | 0.000 | 1.142 | 0.256 | -3.064 | 0.003 |
| **Waist circumference model** | | | | | | | |
|  |  | Tstat_ waist_cir | Pvalue_waist_cir | Tstat_ Age | Pvalue_ Age | Tstat_ Sex | Pvalue_ Sex |
| 1 | precentral | 0.937 | 0.351 | -0.972 | 0.333 | -1.833 | 0.070 |
| 2 | frontal sup | -0.635 | 0.527 | -1.474 | 0.143 | -2.223 | 0.028 |
| 3 | frontal mid | 0.665 | 0.507 | -1.502 | 0.136 | -3.440 | 0.001 |
| 4 | frontal inf oper | 0.596 | 0.552 | -1.429 | 0.156 | -1.470 | 0.144 |
| 5 | frontal inf tri and mid | 0.083 | 0.934 | -3.469 | 0.001 | -2.116 | 0.037 |
| 6 | rolandic oper | 0.159 | 0.874 | -0.835 | 0.406 | -1.016 | 0.312 |
| 7 | sup motor area | -2.951 | 0.004 | -0.446 | 0.657 | -1.116 | 0.267 |
| 8 | olfactory | -2.574 | 0.011 | 0.871 | 0.386 | 0.159 | 0.874 |
| 9 | frontal sup medial | -1.925 | 0.057 | -1.740 | 0.085 | -1.680 | 0.096 |
| 10 | frontal med orb | -2.514 | 0.013 | -1.267 | 0.208 | -1.522 | 0.131 |
| 11 | rectus | -3.619 | 0.000 | -1.192 | 0.236 | 0.274 | 0.784 |
| 12 | OFC | -2.077 | 0.040 | -0.707 | 0.481 | -0.596 | 0.553 |
| 13 | insula | -0.016 | 0.987 | -0.821 | 0.413 | 0.690 | 0.492 |
| 14 | cingulate | -4.543 | 0.000 | -0.176 | 0.860 | -1.341 | 0.183 |
| 15 | hippocampus | -1.540 | 0.126 | -0.073 | 0.942 | -0.359 | 0.720 |
| 16 | parahipoccampal | -1.224 | 0.224 | 0.705 | 0.482 | -1.178 | 0.241 |
| 17 | amygdala | 0.492 | 0.624 | 1.669 | 0.098 | 0.147 | 0.883 |
| 18 | calcarine | -2.714 | 0.008 | 0.021 | 0.983 | -2.268 | 0.025 |
| 19 | cuneus | -1.866 | 0.065 | -0.211 | 0.833 | -1.625 | 0.107 |
| 20 | lingual | -2.671 | 0.009 | -0.054 | 0.957 | -1.234 | 0.220 |
| 21 | occipital | 2.878 | 0.005 | -1.174 | 0.243 | -1.394 | 0.166 |
| 22 | fusiform | -1.506 | 0.135 | -0.545 | 0.587 | -1.090 | 0.278 |
| 23 | postcentral | 1.091 | 0.278 | -0.399 | 0.691 | -0.867 | 0.388 |
| 24 | parietal and supramarginal | 0.746 | 0.457 | -0.199 | 0.843 | -2.109 | 0.037 |
| 25 | angular | 3.221 | 0.002 | -0.782 | 0.436 | -0.309 | 0.758 |
| 26 | precuneus | -4.367 | 0.000 | -0.201 | 0.841 | -2.328 | 0.022 |
| 27 | paracentral lobule | -3.265 | 0.001 | 0.291 | 0.771 | -1.929 | 0.056 |
| 28 | caudate | -2.342 | 0.021 | 0.394 | 0.694 | 0.413 | 0.680 |
| 29 | putamen | 0.423 | 0.673 | 0.492 | 0.624 | 0.457 | 0.648 |
| 30 | pallidum and thalamus | -0.877 | 0.382 | -0.480 | 0.632 | -1.439 | 0.153 |
| 31 | hesch | 0.238 | 0.812 | -0.333 | 0.740 | -0.107 | 0.915 |
| 32 | temporal | 1.097 | 0.275 | 0.124 | 0.901 | 0.146 | 0.884 |
| 33 | cerebellum | -0.769 | 0.444 | 0.157 | 0.876 | -1.620 | 0.108 |
| 34 | vermis | -3.205 | 0.002 | 1.695 | 0.093 | -2.784 | 0.006 |
| **Neck circumference model** | | | | | | | |
|  |  | Tstat_ neck_cir | Pvalue_neck_cir_cir | Tstat_ Age | Pvalue_ Age | Tstat_ Sex | Pvalue_ Sex |
| 1 | precentral | 2.240 | 0.027 | -1.040 | 0.301 | -1.084 | 0.281 |
| 2 | frontal sup | 0.289 | 0.773 | -1.515 | 0.133 | -2.021 | 0.046 |
| 3 | frontal mid | 1.938 | 0.056 | -1.378 | 0.172 | -2.608 | 0.011 |
| 4 | frontal inf oper | 1.901 | 0.060 | -1.567 | 0.120 | -1.206 | 0.231 |
| 5 | frontal inf tri and mid | 1.272 | 0.207 | -3.429 | 0.001 | -1.802 | 0.075 |
| 6 | rolandic oper | 1.258 | 0.211 | -1.262 | 0.210 | -1.044 | 0.299 |
| 7 | sup motor area | -2.475 | 0.015 | -0.573 | 0.568 | -1.462 | 0.147 |
| 8 | olfactory | -1.535 | 0.128 | 0.552 | 0.582 | -0.627 | 0.532 |
| 9 | frontal sup medial | -1.027 | 0.307 | -1.804 | 0.074 | -1.896 | 0.061 |
| 10 | frontal med orb | -1.498 | 0.138 | -1.324 | 0.189 | -1.925 | 0.057 |
| 11 | rectus | -3.172 | 0.002 | -1.584 | 0.117 | -0.936 | 0.352 |
| 12 | OFC | -1.472 | 0.144 | -1.112 | 0.269 | -1.433 | 0.155 |
| 13 | insula | 0.186 | 0.853 | -1.312 | 0.193 | -0.055 | 0.956 |
| 14 | cingulate | -3.206 | 0.002 | -0.700 | 0.486 | -2.424 | 0.017 |
| 15 | hippocampus | -0.797 | 0.428 | -0.783 | 0.435 | -1.239 | 0.219 |
| 16 | parahipoccampal | -0.759 | 0.450 | 0.257 | 0.798 | -1.622 | 0.108 |
| 17 | amygdala | 0.583 | 0.561 | 1.309 | 0.194 | 0.105 | 0.916 |
| 18 | calcarine | -1.587 | 0.116 | -0.326 | 0.745 | -2.857 | 0.005 |
| 19 | cuneus | -1.116 | 0.267 | -0.410 | 0.683 | -1.911 | 0.059 |
| 20 | lingual | -1.532 | 0.129 | -0.249 | 0.804 | -1.646 | 0.103 |
| 21 | occipital | 3.425 | 0.001 | -1.410 | 0.162 | -0.541 | 0.589 |
| 22 | fusiform | 0.117 | 0.907 | -1.243 | 0.217 | -1.572 | 0.119 |
| 23 | postcentral | 2.081 | 0.040 | -0.497 | 0.620 | -0.313 | 0.755 |
| 24 | parietal and supramarginal | 2.055 | 0.043 | -0.391 | 0.697 | -1.527 | 0.130 |
| 25 | angular | 3.901 | 0.000 | -1.069 | 0.288 | 0.479 | 0.633 |
| 26 | precuneus | -2.819 | 0.006 | -0.430 | 0.668 | -2.875 | 0.005 |
| 27 | paracentral lobule | -2.288 | 0.024 | 0.252 | 0.802 | -2.094 | 0.039 |
| 28 | caudate | -1.511 | 0.134 | -0.171 | 0.864 | -0.584 | 0.561 |
| 29 | putamen | 0.631 | 0.529 | -0.232 | 0.817 | 0.028 | 0.978 |
| 30 | pallidum and thalamus | -0.406 | 0.686 | -1.068 | 0.288 | -1.887 | 0.062 |
| 31 | hesch | 0.948 | 0.345 | -0.756 | 0.451 | -0.331 | 0.742 |
| 32 | temporal | 2.276 | 0.025 | -0.214 | 0.831 | 0.283 | 0.778 |
| 33 | cerebellum | 0.524 | 0.601 | -0.227 | 0.821 | -1.583 | 0.117 |
| 34 | vermis | -2.456 | 0.016 | 0.221 | 0.826 | -3.660 | 0.000 |
| **SBP model** | | | | | | | |
|  |  | Tstat_ SBP | Pvalue_ SBP | Tstat _Age | Pvalue _Age | Tstat _Sex | Pvalue _Sex |
| 1 | precentral | 2.086 | 0.039 | -1.079 | 0.283 | -1.909 | 0.058 |
| 2 | frontal sup | 0.830 | 0.408 | -1.271 | 0.206 | -2.145 | 0.034 |
| 3 | frontal mid | 2.479 | 0.014 | -1.353 | 0.178 | -3.259 | 0.001 |
| 4 | frontal inf oper | 2.074 | 0.040 | -1.509 | 0.134 | -1.538 | 0.126 |
| 5 | frontal inf tri and mid | 1.448 | 0.150 | -3.443 | 0.001 | -2.054 | 0.042 |
| 6 | rolandic oper | 1.658 | 0.100 | -0.866 | 0.388 | -1.110 | 0.269 |
| 7 | sup motor area | -2.060 | 0.041 | -0.073 | 0.942 | -0.979 | 0.330 |
| 8 | olfactory | -1.193 | 0.235 | 1.260 | 0.210 | 0.217 | 0.829 |
| 9 | frontal sup medial | -0.356 | 0.723 | -1.295 | 0.198 | -1.490 | 0.139 |
| 10 | frontal med orb | -0.738 | 0.462 | -0.684 | 0.495 | -1.356 | 0.177 |
| 11 | rectus | -2.509 | 0.013 | -0.765 | 0.446 | 0.199 | 0.842 |
| 12 | OFC | -1.498 | 0.137 | -0.540 | 0.590 | -0.805 | 0.422 |
| 13 | insula | 0.422 | 0.674 | -0.826 | 0.411 | 0.625 | 0.533 |
| 14 | cingulate | -2.451 | 0.016 | 0.296 | 0.768 | -1.218 | 0.225 |
| 15 | hippocampus | -0.166 | 0.868 | -0.065 | 0.949 | -0.427 | 0.670 |
| 16 | parahipoccampal | -0.569 | 0.570 | 0.823 | 0.412 | -1.144 | 0.255 |
| 17 | amygdala | 0.386 | 0.700 | 1.752 | 0.082 | 0.199 | 0.843 |
| 18 | calcarine | -2.006 | 0.047 | 0.218 | 0.827 | -2.368 | 0.019 |
| 19 | cuneus | -1.164 | 0.247 | 0.005 | 0.996 | -1.548 | 0.124 |
| 20 | lingual | -2.017 | 0.046 | 0.289 | 0.773 | -1.162 | 0.248 |
| 21 | occipital | 3.552 | 0.001 | -1.405 | 0.162 | -1.308 | 0.193 |
| 22 | fusiform | -0.224 | 0.823 | -0.594 | 0.554 | -1.193 | 0.235 |
| 23 | postcentral | 2.183 | 0.031 | -0.429 | 0.669 | -0.767 | 0.444 |
| 24 | parietal and supramarginal | 1.956 | 0.053 | -0.214 | 0.831 | -2.169 | 0.032 |
| 25 | angular | 4.238 | 0.000 | -1.225 | 0.223 | -0.379 | 0.705 |
| 26 | precuneus | -2.821 | 0.006 | 0.362 | 0.718 | -2.170 | 0.032 |
| 27 | paracentral lobule | -2.713 | 0.008 | 0.679 | 0.499 | -1.742 | 0.084 |
| 28 | caudate | -0.966 | 0.336 | 0.516 | 0.606 | 0.429 | 0.669 |
| 29 | putamen | 0.475 | 0.636 | 0.513 | 0.609 | 0.198 | 0.843 |
| 30 | pallidum and thalamus | 0.118 | 0.906 | -0.572 | 0.568 | -1.565 | 0.120 |
| 31 | hesch | 1.177 | 0.241 | -0.353 | 0.724 | -0.159 | 0.874 |
| 32 | temporal | 2.251 | 0.026 | 0.124 | 0.902 | 0.059 | 0.953 |
| 33 | cerebellum | 0.150 | 0.881 | 0.220 | 0.826 | -1.533 | 0.128 |
| 34 | vermis | -2.168 | 0.032 | 1.525 | 0.130 | -3.060 | 0.003 |
| **TG model** | | | | | | | |
|  |  | Tstat _TG | Pvalue_ TG | Tstat_ Age | Pvalue_ Age | Tstat_ Sex | Pvalue_ Sex |
| 1 | precentral | 1.760 | 0.081 | -1.094 | 0.276 | -1.805 | 0.074 |
| 2 | frontal sup | 0.540 | 0.590 | -1.329 | 0.186 | -2.138 | 0.035 |
| 3 | frontal mid | 1.298 | 0.197 | -1.613 | 0.109 | -3.420 | 0.001 |
| 4 | frontal inf oper | -0.060 | 0.952 | -1.884 | 0.062 | -1.657 | 0.100 |
| 5 | frontal inf tri and mid | -0.257 | 0.797 | -3.701 | 0.000 | -2.154 | 0.033 |
| 6 | rolandic oper | -0.365 | 0.716 | -0.977 | 0.330 | -1.107 | 0.270 |
| 7 | sup motor area | -1.237 | 0.219 | 0.308 | 0.758 | -0.522 | 0.603 |
| 8 | olfactory | -2.585 | 0.011 | 1.363 | 0.175 | 0.495 | 0.622 |
| 9 | frontal sup medial | -0.860 | 0.392 | -1.295 | 0.198 | -1.393 | 0.166 |
| 10 | frontal med orb | -0.788 | 0.433 | -0.709 | 0.480 | -1.254 | 0.212 |
| 11 | rectus | -2.584 | 0.011 | -0.647 | 0.519 | 0.534 | 0.595 |
| 12 | OFC | -1.904 | 0.059 | -0.505 | 0.614 | -0.580 | 0.563 |
| 13 | insula | -1.305 | 0.194 | -0.594 | 0.554 | 0.770 | 0.443 |
| 14 | cingulate | -2.729 | 0.007 | 0.381 | 0.704 | -0.926 | 0.357 |
| 15 | hippocampus | -1.279 | 0.203 | 0.007 | 0.994 | -0.191 | 0.849 |
| 16 | parahipoccampal | -1.673 | 0.097 | 0.773 | 0.441 | -1.019 | 0.310 |
| 17 | amygdala | -0.771 | 0.442 | 1.695 | 0.093 | 0.231 | 0.818 |
| 18 | calcarine | -2.158 | 0.033 | 0.311 | 0.756 | -2.183 | 0.031 |
| 19 | cuneus | -1.557 | 0.122 | 0.029 | 0.977 | -1.409 | 0.161 |
| 20 | lingual | -2.812 | 0.006 | 0.242 | 0.809 | -0.997 | 0.321 |
| 21 | occipital | 1.008 | 0.315 | -1.514 | 0.133 | -1.321 | 0.189 |
| 22 | fusiform | -1.894 | 0.061 | -0.657 | 0.513 | -1.018 | 0.311 |
| 23 | postcentral | 0.784 | 0.434 | -0.564 | 0.574 | -0.735 | 0.464 |
| 24 | parietal and supramarginal | 1.647 | 0.102 | -0.093 | 0.926 | -2.154 | 0.033 |
| 25 | angular | 2.741 | 0.007 | -1.389 | 0.167 | -0.569 | 0.570 |
| 26 | precuneus | -2.216 | 0.029 | 0.644 | 0.521 | -1.747 | 0.083 |
| 27 | paracentral lobule | -1.764 | 0.080 | 1.060 | 0.291 | -1.343 | 0.182 |
| 28 | caudate | -1.479 | 0.142 | 0.601 | 0.549 | 0.694 | 0.489 |
| 29 | putamen | 0.778 | 0.438 | 0.626 | 0.533 | 0.318 | 0.751 |
| 30 | pallidum and thalamus | -0.470 | 0.639 | -0.469 | 0.640 | -1.341 | 0.183 |
| 31 | hesch | -0.650 | 0.517 | -0.504 | 0.615 | -0.177 | 0.859 |
| 32 | temporal | 0.606 | 0.546 | -0.020 | 0.984 | -0.041 | 0.967 |
| 33 | cerebellum | -0.532 | 0.595 | 0.223 | 0.824 | -1.477 | 0.142 |
| 34 | vermis | -2.599 | 0.011 | 1.729 | 0.086 | -3.049 | 0.003 |
| **Insulin model** | | | | | | | |
|  |  | Tstat_ Insulin | Pvalue_ Insulin | Tstat_ Age | Pvalue_ Age | Tstat_ Sex | Pvalue_ Sex |
| 1 | precentral | 0.299 | 0.766 | -0.859 | 0.392 | -1.538 | 0.127 |
| 2 | frontal sup | -1.270 | 0.207 | -1.407 | 0.162 | -2.106 | 0.037 |
| 3 | frontal mid | 0.399 | 0.691 | -1.376 | 0.172 | -3.145 | 0.002 |
| 4 | frontal inf oper | 0.572 | 0.568 | -1.433 | 0.155 | -1.382 | 0.170 |
| 5 | frontal inf tri and mid | -0.083 | 0.934 | -3.404 | 0.001 | -1.980 | 0.050 |
| 6 | rolandic oper | 0.093 | 0.926 | -0.691 | 0.491 | -0.877 | 0.382 |
| 7 | sup motor area | -3.473 | 0.001 | 0.008 | 0.994 | -0.716 | 0.475 |
| 8 | olfactory | -1.204 | 0.231 | 1.361 | 0.176 | 0.386 | 0.701 |
| 9 | frontal sup medial | -2.164 | 0.033 | -1.459 | 0.147 | -1.473 | 0.144 |
| 10 | frontal med orb | -2.038 | 0.044 | -0.900 | 0.370 | -1.365 | 0.175 |
| 11 | rectus | -3.574 | 0.001 | -0.971 | 0.334 | 0.240 | 0.811 |
| 12 | OFC | -2.267 | 0.025 | -0.600 | 0.550 | -0.644 | 0.521 |
| 13 | insula | -0.311 | 0.756 | -0.534 | 0.594 | 0.792 | 0.430 |
| 14 | cingulate | -3.051 | 0.003 | 0.222 | 0.824 | -1.105 | 0.272 |
| 15 | hippocampus | -1.625 | 0.107 | -0.047 | 0.962 | -0.303 | 0.762 |
| 16 | parahipoccampal | -1.263 | 0.209 | 0.802 | 0.424 | -1.050 | 0.296 |
| 17 | amygdala | 0.036 | 0.971 | 1.907 | 0.059 | 0.444 | 0.658 |
| 18 | calcarine | -1.581 | 0.117 | 0.308 | 0.759 | -2.213 | 0.029 |
| 19 | cuneus | -1.275 | 0.205 | 0.070 | 0.944 | -1.448 | 0.150 |
| 20 | lingual | -1.721 | 0.088 | 0.296 | 0.768 | -1.020 | 0.310 |
| 21 | occipital | 1.310 | 0.193 | -1.157 | 0.250 | -1.053 | 0.295 |
| 22 | fusiform | -1.718 | 0.089 | -0.649 | 0.518 | -1.064 | 0.290 |
| 23 | postcentral | 0.419 | 0.676 | -0.214 | 0.831 | -0.480 | 0.632 |
| 24 | parietal and supramarginal | 0.321 | 0.749 | 0.174 | 0.862 | -1.812 | 0.073 |
| 25 | angular | 2.214 | 0.029 | -0.947 | 0.346 | -0.149 | 0.882 |
| 26 | precuneus | -2.890 | 0.005 | 0.409 | 0.683 | -1.926 | 0.057 |
| 27 | paracentral lobule | -3.102 | 0.002 | 0.860 | 0.392 | -1.528 | 0.129 |
| 28 | caudate | -1.285 | 0.201 | 0.642 | 0.522 | 0.666 | 0.507 |
| 29 | putamen | 0.244 | 0.808 | 0.781 | 0.436 | 0.562 | 0.575 |
| 30 | pallidum and thalamus | -0.626 | 0.532 | -0.558 | 0.578 | -1.419 | 0.159 |
| 31 | hesch | -0.301 | 0.764 | -0.253 | 0.801 | -0.006 | 0.995 |
| 32 | temporal | 0.370 | 0.712 | 0.266 | 0.791 | 0.254 | 0.800 |
| 33 | cerebellum | -1.195 | 0.235 | 0.423 | 0.673 | -1.417 | 0.159 |
| 34 | vermis | -2.281 | 0.024 | 1.548 | 0.124 | -3.228 | 0.002 |
| **HOMA-IR model** | | | | | | | |
|  |  | Tstat_ HOMAIR | Pvalue_ HOMAIR | Tstat_ Age | Pvalue_ Age | Tstat_ Sex | Pvalue_ Sex |
| 1 | precentral | 0.309 | 0.758 | -0.861 | 0.391 | -1.543 | 0.126 |
| 2 | frontal sup | -1.289 | 0.200 | -1.400 | 0.164 | -2.092 | 0.039 |
| 3 | frontal mid | 0.252 | 0.801 | -1.406 | 0.162 | -3.163 | 0.002 |
| 4 | frontal inf oper | 0.345 | 0.731 | -1.491 | 0.139 | -1.410 | 0.161 |
| 5 | frontal inf tri and mid | -0.376 | 0.708 | -3.456 | 0.001 | -2.002 | 0.048 |
| 6 | rolandic oper | -0.093 | 0.926 | -0.716 | 0.475 | -0.892 | 0.374 |
| 7 | sup motor area | -3.313 | 0.001 | 0.073 | 0.942 | -0.660 | 0.510 |
| 8 | olfactory | -1.550 | 0.124 | 1.349 | 0.180 | 0.382 | 0.703 |
| 9 | frontal sup medial | -2.247 | 0.027 | -1.450 | 0.150 | -1.452 | 0.149 |
| 10 | frontal med orb | -1.964 | 0.052 | -0.863 | 0.390 | -1.333 | 0.185 |
| 11 | rectus | -3.529 | 0.001 | -0.910 | 0.365 | 0.283 | 0.777 |
| 12 | OFC | -2.441 | 0.016 | -0.592 | 0.555 | -0.626 | 0.533 |
| 13 | insula | -0.373 | 0.710 | -0.530 | 0.597 | 0.795 | 0.428 |
| 14 | cingulate | -3.007 | 0.003 | 0.270 | 0.788 | -1.051 | 0.295 |
| 15 | hippocampus | -1.604 | 0.112 | -0.027 | 0.979 | -0.278 | 0.782 |
| 16 | parahipoccampal | -1.305 | 0.195 | 0.811 | 0.419 | -1.036 | 0.302 |
| 17 | amygdala | -0.217 | 0.829 | 1.884 | 0.062 | 0.428 | 0.669 |
| 18 | calcarine | -1.785 | 0.077 | 0.297 | 0.767 | -2.212 | 0.029 |
| 19 | cuneus | -1.436 | 0.154 | 0.057 | 0.955 | -1.451 | 0.150 |
| 20 | lingual | -1.893 | 0.061 | 0.289 | 0.773 | -1.016 | 0.312 |
| 21 | occipital | 1.229 | 0.222 | -1.181 | 0.240 | -1.073 | 0.286 |
| 22 | fusiform | -1.730 | 0.086 | -0.630 | 0.530 | -1.040 | 0.301 |
| 23 | postcentral | 0.264 | 0.792 | -0.244 | 0.808 | -0.496 | 0.621 |
| 24 | parietal and supramarginal | 0.449 | 0.654 | 0.188 | 0.851 | -1.812 | 0.073 |
| 25 | angular | 2.234 | 0.027 | -0.961 | 0.338 | -0.166 | 0.869 |
| 26 | precuneus | -2.951 | 0.004 | 0.443 | 0.658 | -1.882 | 0.062 |
| 27 | paracentral lobule | -3.013 | 0.003 | 0.910 | 0.365 | -1.481 | 0.141 |
| 28 | caudate | -1.415 | 0.160 | 0.650 | 0.517 | 0.675 | 0.501 |
| 29 | putamen | 0.278 | 0.782 | 0.777 | 0.439 | 0.559 | 0.577 |
| 30 | pallidum and thalamus | -0.940 | 0.349 | -0.556 | 0.580 | -1.425 | 0.157 |
| 31 | hesch | -0.359 | 0.721 | -0.258 | 0.797 | -0.007 | 0.995 |
| 32 | temporal | 0.195 | 0.846 | 0.249 | 0.804 | 0.241 | 0.810 |
| 33 | cerebellum | -1.248 | 0.215 | 0.435 | 0.665 | -1.394 | 0.166 |
| 34 | vermis | -2.286 | 0.024 | 1.592 | 0.114 | -3.220 | 0.002 |
| **CRP Model** | | | | | | | |
|  |  | Tstat_ CRP | Pvalue_ CRP | Tstat_ Age | Pvalue_ Age | Tstat_ Sex | Pvalue_ Sex |
| 1 | precentral | -0.690 | 0.495 | -0.630 | 0.528 | 0.400 | 0.690 |
| 2 | frontal sup | -0.040 | 0.966 | -2.030 | 0.048 | 0.830 | 0.408 |
| 3 | frontal mid | 0.520 | 0.608 | -2.470 | 0.017 | 2.760 | 0.008 |
| 4 | frontal inf oper | -0.210 | 0.836 | -3.450 | 0.001 | 2.450 | 0.017 |
| 5 | frontal inf tri and mid | 0.760 | 0.448 | -4.690 | 0.0001 | 0.880 | 0.385 |
| 6 | rolandic oper | -0.410 | 0.685 | -2.380 | 0.021 | 0.560 | 0.576 |
| 7 | sup motor area | -1.060 | 0.293 | -0.550 | 0.587 | -0.390 | 0.701 |
| 8 | olfactory | 0.090 | 0.930 | -0.960 | 0.341 | -0.020 | 0.983 |
| 9 | frontal sup medial | 0.210 | 0.832 | -2.800 | 0.007 | 0.570 | 0.574 |
| 10 | frontal med orb | -0.160 | 0.874 | -1.390 | 0.1700 | 0.720 | 0.477 |
| 11 | rectus | -0.930 | 0.358 | -2.850 | 0.006 | -0.830 | 0.410 |
| 12 | OFC | 0.740 | 0.462 | -3.270 | 0.002 | -0.360 | 0.719 |
| 13 | insula | -0.420 | 0.680 | -2.040 | 0.047 | -1.620 | 0.112 |
| 14 | cingulate | -1.660 | 0.104 | -2.980 | 0.004 | 1.840 | 0.071 |
| 15 | hippocampus | -0.620 | 0.539 | -0.010 | 0.991 | -0.140 | 0.887 |
| 16 | parahipoccampal | 0.540 | 0.593 | 0.080 | 0.938 | 0.110 | 0.910 |
| 17 | amygdala | 0.440 | 0.663 | 0.620 | 0.537 | 0.390 | 0.699 |
| 18 | calcarine | 0.220 | 0.825 | -1.080 | 0.283 | 2.070 | 0.043 |
| 19 | cuneus | 0.110 | 0.910 | -0.160 | 0.872 | 1.190 | 0.240 |
| 20 | lingual | -0.080 | 0.937 | -1.880 | 0.066 | 1.400 | 0.166 |
| 21 | occipital | 0.360 | 0.720 | -2.510 | 0.015 | 1.550 | 0.127 |
| 22 | fusiform | 0.000 | 0.997 | -1.360 | 0.178 | -0.060 | 0.954 |
| 23 | postcentral | 0.070 | 0.947 | -1.050 | 0.301 | 0.280 | 0.780 |
| 24 | parietal and supramarginal | -1.220 | 0.229 | -1.360 | 0.176 | 1.840 | 0.072 |
| 25 | angular | 0.720 | 0.476 | -2.910 | 0.005 | 0.110 | 0.913 |
| 26 | precuneus | -1.790 | 0.079 | -2.570 | 0.013 | 2.480 | 0.017 |
| 27 | paracentral lobule | -1.930 | 0.059 | 0.300 | 0.763 | 0.750 | 0.457 |
| 28 | caudate | -1.560 | 0.125 | -1.040 | 0.301 | 0.070 | 0.941 |
| 29 | putamen | -1.710 | 0.093 | 0.220 | 0.828 | -0.250 | 0.803 |
| 30 | pallidum and thalamus | 0.510 | 0.612 | -0.440 | 0.665 | 0.800 | 0.426 |
| 31 | hesch | -0.490 | 0.626 | -1.650 | 0.106 | -0.290 | 0.772 |
| 32 | temporal | -0.420 | 0.675 | -0.820 | 0.415 | -0.120 | 0.908 |
| 33 | cerebellum | -0.290 | 0.773 | -0.620 | 0.540 | 2.040 | 0.046 |
| 34 | vermis | -0.360 | 0.720 | 1.150 | 0.255 | 1.710 | 0.093 |

Results are presented as T-stat and p-value. Model used: Adjusted VBM changes ∼ changes in adiposity/metabolic/inflammatory variables + Age + Sex, BMI, body mass index; TWL, total weight loss; SBP, systolic blood pressure; TG, triglycerides levels; HOMA-IR, homeostasis model assessment of insulin resistance; CRP, C-Reactive Protein
